# Evidence for holocentric centromeres in the early branching apicomplexan parasite *Cryptosporidium parvum*

**DOI:** 10.1371/journal.ppat.1014708

**Published:** 2026-10-26

**Authors:** Abigail Kimball, Wanyi Huang, Melanie Key, Rui Xu, Lisa Funkhouser-Jones, L. David Sibley

**Affiliations:** Department of Molecular Microbiology, Washington University in St. Louis, St. Louis, Missouri, United States of America; The Walter and Eliza Hall Institute of Medical Research, AUSTRALIA

## Abstract

During its asexual cycle, *Cryptosporidium parvum* (*C. parvum*) amplifies through three rounds of nuclear division before undergoing cytokinesis to form infectious merozoites. However, chromosome organization and segregation during nuclear division remain poorly studied. Here, we visualized H3-histones, including the centromeric histone H3 (CENH3), telomeres, and centrosomes during mitosis. Nuclear division was accompanied by centrosome duplication and elongated microtubules that spanned the dividing nuclei. Surprisingly, *C. parvum* centromeres detected by CENH3 showed diffuse staining throughout nuclear division and overlapped with telomeres in the apical nucleus. Analysis of centromeres based on CENH3 capture and DNA sequencing revealed numerous distinct binding sites scattered across all eight chromosomes, including both coding regions and intergenic regions, which were typified by GA rich repeat sequences. The unique holocentric chromosome structure of *C. parvum* is unlike the single regional centromeres found in related apicomplexans, suggesting it arose independently from other known examples in plants, insects and worms.

## Author summary

*Cryptosporidium parvum* is a widespread cause of diarrheal disease worldwide, and lack of knowledge about its basic biology has hindered progress to develop more effective treatments. Unlike most organisms, *C. parvum* divides its chromosomes within an intact nucleus and during this process, its chromosomes remain in a relaxed, uncondensed state, making the division processes difficult to visualize. Here, we used advanced imaging and genomic fingerprinting to examine how chromosomes are positioned and segregated during replication. We discovered that instead of having a single attachment point, known as the centromere, on each chromosome, *C. parvum* distributes hundreds of small attachment sites along all eight chromosomes. These dispersed sites remain diffuse throughout mitosis and overlap with telomeres at the chromosomes ends, revealing a chromosome organization known as holocentricity. This architecture has been described in certain plants, insects, and nematodes but has not previously been identified in protists. By defining this unusual centromere architecture, our work provides new insight into how *C. parvum* maintains genome stability during rapid, repeated rounds of nuclear division.

## Introduction

*Cryptosporidium parvum (C. parvum)*, an obligate intracellular parasite, remains a serious global health and agricultural threat [1–4]. Transmission occurs through the ingestion of infectious oocysts and causes severe diarrheal illness in infants, young animals, and immunocompromised adults [5–7]. Once ingested, sporozoites exit the oocyst, invade host cells, and undergo three rounds of nuclear division within a single shared cytoplasm (the process of merogony), followed by cytokinesis to produce eight identical merozoites. Newly formed merozoites are released into the lumen, where they invade new host cells, and repeat the invasion-egress cycle three times before transitioning to the sexual phase of the life cycle [8,9]. Despite its significance as a global health problem, and the novelty of apicomplexan nuclear division processes, *C. parvum* mitosis remains largely uncharacterized, with our current understanding limited to basic microscopy studies [8,10–15].

To divide rapidly, apicomplexans appear to rely on several features that are thought to streamline nuclear division. Comparative cell biology suggests that maintaining chromosomes in a minimally condensed state may reduce the need for extensive chromatin remodeling during division, although the extent to which this directly accelerates mitosis remains to be fully resolved [16,17]. This adaptation necessitates strict nuclear organization during mitosis, where centromeres are typically maintained on the apical end of the nucleus attached to the centrosomes, and telomeres persistently cluster at the basal end of the nuclear periphery [18–20]. Additionally, apicomplexans maintain their nuclear envelope throughout mitosis, minimizing the energy and time required for breakdown and reformation [17,21]. As a consequence of this closed mitosis apicomplexan centrosomes (spindle pole bodies, microtubule-organizing centers) are embedded within the nuclear envelope and serve as spindle assembly hubs that elongate the microtubules so they can attach to the centromeres and ultimately coordinate chromosome movement during division [22–29]. It has been postulated that nuclear pores are essential for nucleocytoplasmic transport through the intact envelope and directly support the process of spindle assembly [30,31]. Electron microscopy studies suggest that *C. parvum* undergoes schizogony, with multiple rounds of nuclear replication preceding a cytoplasmic division, followed by the egress of daughter parasites from the host cell [13].

During mitosis and meiosis, centromeres function to orchestrate chromosome segregation by serving as sites for microtubule anchoring to assure faithful separation at metaphase [32]. Centromeres have variable architecture in different organisms consisting of point centromeres (small 250 bp single site per chromosome) in yeast [33], regional centromeres (gene poor islands of heterochromatin ranging from several to hundreds of kb) in *T. gondii* and *P. falciparum* [26,27,34], and holocentric chromosomes (multiple centromere sites along the chromosome) found in multiple species of plants, insects and worms [35–37]. Although centromere function is essential and highly conserved across eukaryotes, centromeric DNA sequences and size are subject to rapid evolution [38–40]. Variation in centromere architecture affects nuclear organization, karyotype changes, and ultimately drives speciation [41,42].

The nuclear structures, chromosome features, and mitotic mechanisms, including the role of centromeres, have not been examined previously in *C. parvum*. Here, we delineate the fundamental biology of *C. parvum* mitosis, revealing significant deviations from previously assumed mechanisms. Using high-resolution imaging we examined nuclear division during the first round of merogony, leveraging multiple transgenic parasite strains and mitosis-specific antibodies. Additionally, we used Cleavage Under Targets and Nuclease Release (CUT&RUN) [43] to identify the location of the centromeres based on CENH3 and patterns of centromere-associated histone modifications in *C. parvum* and the related parasite *Toxoplasma gondii* (*T. gondii*). In contrast to the monocentric centromeric structure of *T. gondii*, we found that *C. parvum* has a dispersed centromeric chromosome structure with CENH3 binding sites scattered across multiple small regions on all 8 chromosomes, resulting in diffuse CENH3 centromere staining throughout mitosis. Overall, these results suggest that *C. parvum* employs a divergent mitotic mechanism compared to other well-studied apicomplexans and has convergently evolved holocentric architecture.

## Results

### Characterization of centromeric histone H3 (CENH3) and histone H3 candidates in *C. parvum*

The genome annotation of *C. parvum* contains two genes encoding Histone H3 *cgd3_2540* and *cgd4_3220* (referred to here as H3.1 and H3.2, respectively) and a third gene *cgd4_2030* annotated as a centromere-specific Histone H3 variant (CENH3). To validate these annotations, we performed multiple BLASTP analyses using the protein products of each of these three *C. parvum* genes to identify top hits in the following groups: 1) Apicomplexa (excluding all *Cryptosporidium* species), 2) all eukaryotic organisms (excluding all Apicomplexa), 3) *Homo sapiens*, and 4) *Saccharomyces cerevisiae.* Collectively, these analyses provide strong support for cgd4_2030 as the CENH3 ortholog in *C. parvum* (Table 1). To examine the relationship between the protein sequences for the *C. parvum* genes of interest and the top hits of BLASTP analyses, a phylogenetic tree was generated (Fig 1A). We found that *cgd3_2540* (H3.1) and *cgd4_3220* (H3.2) closely cluster with histone H3 sequences from other organisms, whereas *cgd4_2030* (CENH3) clustered with other centromeric histone H3 proteins. We then directly compared the amino acid sequences of H3.1 and H3.2 with a global sequence alignment and found that the two sequences were highly similar (99.26%), but less similar compared to the CENH3 (65.10% and 64.43%, respectively) (Table 2 and Fig 1B).

**Table 1:** The results of BLASTP analyses using the *C. parvum* centromeric histone H3-like protein (*cgd4_2030*), histone H3-like proteins (*cgd3_2540* and *cgd4_3220).*

Query	Top hits in Apicomplexa^*^	E value	Top hits in all Eukaryotes^#^	E value	Top hits in *Saccharomyces cerevisiae*	E value	Top hits in *Homo sapiens*	E value
*cgd4_2030* (CENH3)	histone H3-like centromeric protein CSE4 [*C. cionae*]	5e-46	centromeric DNA-binding histone H3-like protein cse4 [*N. ribis*]	3e-39	Chain A, Cse4	2e-30	Chain A, Histone H3.1, Histone H3-like centromeric protein A, Histone H3.1	5e-39
*cgd3_2540* (H3.1)	histone H3 [*C. cionae*]	2e-86	histone H3-like [*P. hypophthalmus*]	3e-86	histone H3	4e-81	histone H3.2	1e-88
*cgd4_3220* (H3.2)	histone H3 [*C. cionae*]	2e-90	histone H3-like [*P. hypophthalmus*]	2e-87	histone H3	2e-81	histone H3.2	1e-89

* *C. parvum* proteins were compared against all organisms in the Apicomplexa phylum (except for *Cryptosporidium* spp.),

# *C. parvum* proteins were compared against all organisms (except for Apicomplexa).

**Table 2:** The identity and similarity results from multiple global sequence alignments between H3.1, H3.2, and CENH3 proteins using a BLOSUM62 scoring matrix.

*Cp* H3.1 and *Cp* H3.2	*Cp* CENH3 and *Cp* H3.1	*Cp* CENH3 and *Cp* H3.2
	Identity: 46.31% Similarity: 65.10% Gaps: 14.09%	
Identity: 97.78% Similarity: 99.26% Gaps: 0.00%		Identity: 46.31% Similarity: 64.43% Gaps: 14.09%

**Fig 1:**
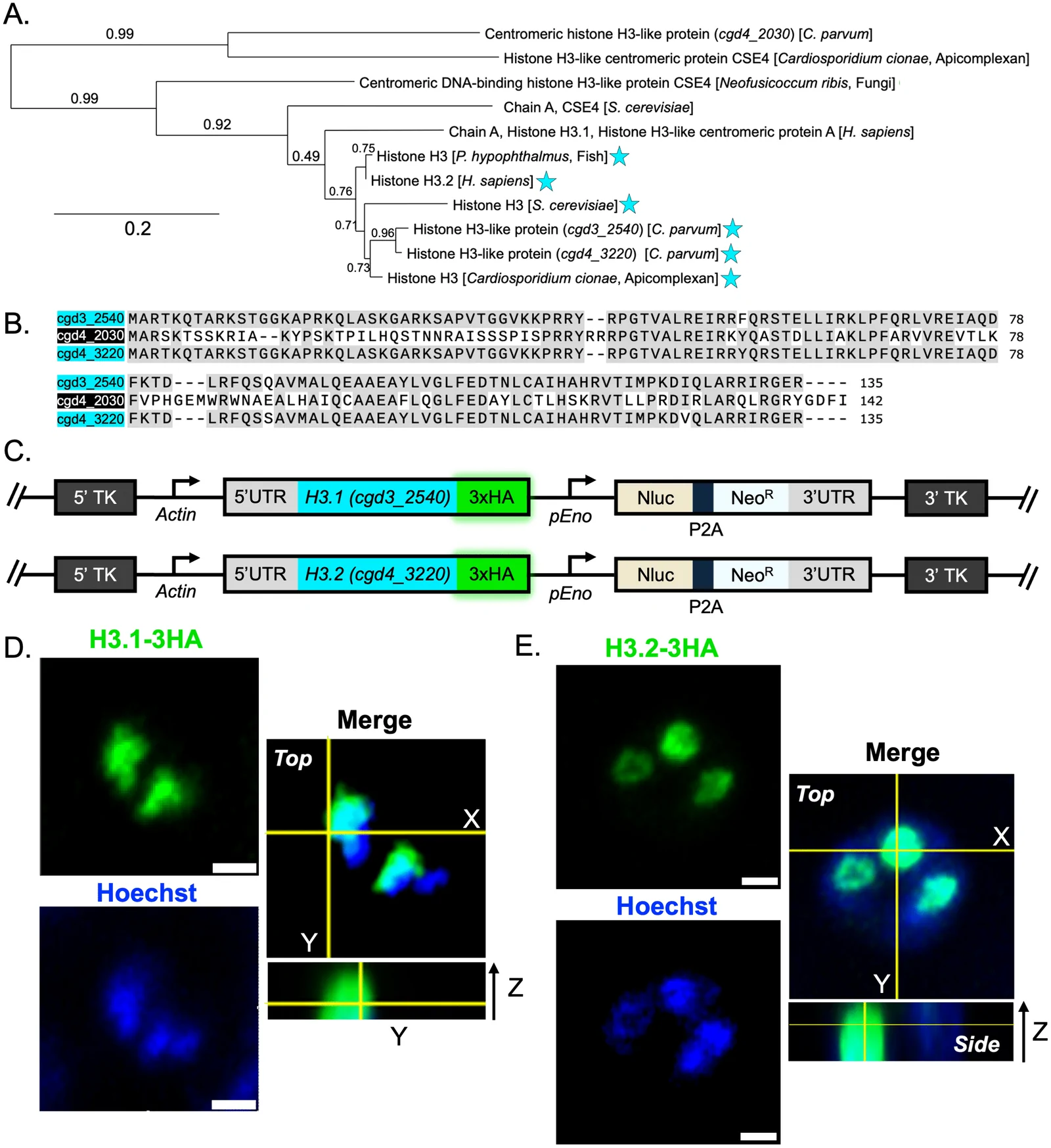
Characterization of centromeric histone H3 (CENH3) and histone H3 candidates in *C. parvum* and construction of Histone H3-3HA tagged strains. **(A)** Phylogenetic relationship of *C. parvum* H3 and centromeric H3 (CENH3) histones and their corresponding top BLASTP hits in other eukaryotes, apicomplexan parasites, *Saccharomyces cerevisiae*, or *Homo sapiens*. Cyan stars represent annotated histone H3 genes. **(B)** Protein sequence alignment of H3 (cyan) and CENH3 (black) in *C. parvum*. Amino acids that are identical between at least two sequences are highlighted in grey. **(C)** Diagram of the targeting constructs designed to replace the endogenous *tk* locus with a second copy of either of the genes annotated as Histone H3-like proteins in *C. parvum* (*cgd3_2540* or *cgd4_3220*), a 3HA tag, and a Nluc-P2A-NeoR cassette. **(D)** Immunofluorescence staining of transgenic H3.1-3HA or **(E)** H3.2-3HA parasites. HCT-8 cells were infected with transgenic oocysts, fixed at 18 hpi, and stained with rat anti-HA followed by the secondary antibody Alexa Fluor 488 goat anti-rat IgG. Hoechst was used for nuclear staining. Images were acquired as Z-stacks using a Zeiss LSM-880 Laser Scanning Confocal microscope equipped with Airyscan (LSCM-A) and are presented with orthogonal views. Scale bars, 1 μm. Data represent two biological replicates, each with 3-5 technical replicates.

To further explore the distribution of H3.1 and H3.2 H3 variants, we generated two transgenic lines of parasites that replaced the dispensable thymidine kinase (*tk*) gene with a second copy of *cgd3_2540* (referred to as H3.1-3HA) or *cgd4_3220* (referred to as H3.2-3HA) fused with a triple hemagglutinin (3HA) epitope tag using CRISPR/Cas9 (Fig 1C, S1A-S1B Fig, S1 Table). Transgenic parasites were used to infect a human adenocarcinoma cell line (HCT-8) monolayer, cultures were fixed at 18 hours post infection (hpi) during merogony, stained, and the localization of the 3HA tag was observed by immunofluorescence (IFA) high-resolution confocal laser scanning microscopy equipped with Airyscan detection (LSCM-A). We found that H3.1-3HA (Fig 1D) and H3.2-3HA (Fig 1E) had a diffuse staining pattern, which was expected based on histone H3 staining in other apicomplexan parasites [27,44,45]

### The staining pattern of *C. parvum* centromeric histone H3 (CENH3) is distinct

To visualize *C. parvum* centromeres, we used CRISPR-Cas9 gene editing to add a 3HA tag to the C-terminus of the centromere-specific histone H3 variant protein (referred to as CENH3-3HA) (Fig 2A, S2A-B Fig, S1 Table). After successfully selecting and amplifying these transgenic parasites in immunocompromised mice, we infected HCT-8 cells for 22 h and examined the staining pattern by LSCM-A microscopy. For these studies, we chose mature meronts that were oriented with the basal side of the vacuole at the surface of the host cell where it attaches at the feeder organelle and where the majority of meronts were projecting upward, as ascertained by fluorescence staining and Z-stack images running perpendicular from bottom to top of the parasite (see cartoon on Fig 2C). The CENH3 staining pattern observed in *C. parvum* was unexpectedly highly diffuse and occupied the apical half of the nucleus (Fig 2B-C) in contrast to the more dispersed pattern found in the H3.1-3HA and H3.2-3HA tagged parasites (Fig 1D, 1E). The diffuse pattern of CENH3 staining in *C. parvum* contrasts sharply with the focal pattern of regional centromeres previously described in *T. gondii* [27,46]. Hence, we utilized a CENH3-3HA-tagged *T. gondii*, gifted by Dr. Kami Kim, as a staining control for our experiments. These parasites were used for the infection of HCT-8 cells, fixed at 24 hpi, stained, and examined by high-resolution LSCM-A immunofluorescence (IFA) microscopy (Fig 2D-E). We found that the staining pattern of the centromeric histone H3 in *T. gondii* is punctate, similar to what has been found previously in *T. gondii* by other investigators [27,46].

**Fig 2:**
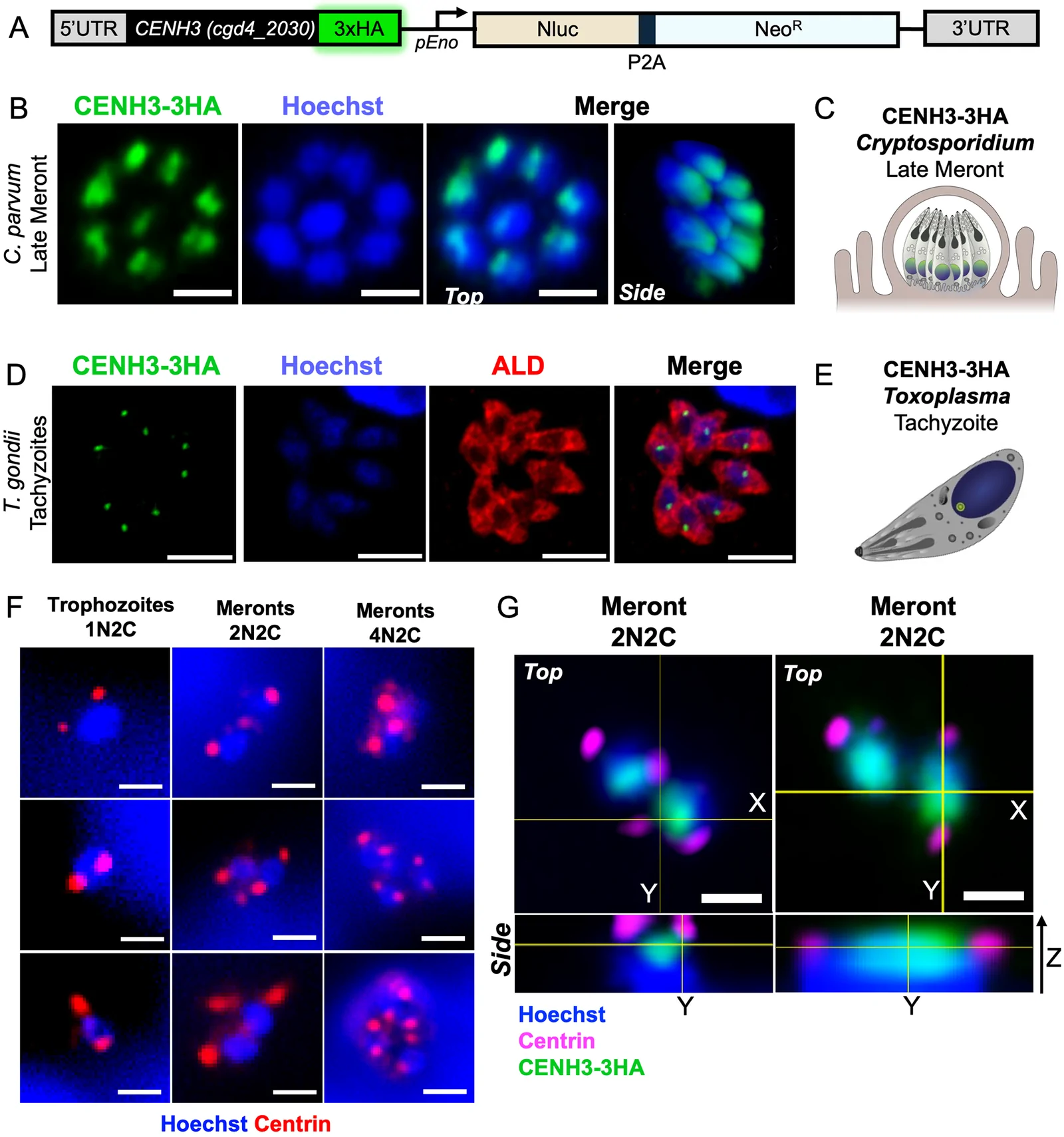
The staining pattern of *C. parvum* centromeric histone H3 (CENH3) is distinct from *Toxoplasma* and remains diffuse during mitosis. **(A)** Diagram of the targeting construct designed to add a 3HA tag and Nluc-P2A-Neo^R^ cassette to the C-terminus of the *C. parvum* CENH3 (*cgd4_2030*). **(B)** HCT-8 cells were infected with CENH3-3HA *C. parvum* parasites, fixed at 22 hpi, and stained with rat anti-HA, followed by the secondary antibody Alexa Fluor 488 goat anti-rat IgG followed by Hoechst staining. Images were acquired as Z-stacks using LSCM-A and are presented with orthogonal views. Scale bars, 1 μm. **(C)** Cartoon depiction corresponding to the microscopy image in panel **(B)**. In *Cryptosporidium*, a late-stage meront contains eight nuclei that have recently completed cytokinesis to form mature merozoites. The CENH3-3HA signal within each nucleus displays a diffuse staining pattern in *C. parvum*. **(D)** HCT-8 cells were infected with CENH3-3HA *T. gondii* parasites, fixed at 24 hpi, and stained with rat anti-HA and rabbit anti-aldolase (ALD) to visualize the cytosol, followed by secondary antibodies Alexa Fluor 488 goat anti-rat IgG and Alexa Fluor 568 anti-rabbit IgG. Hoechst was used for nuclear staining. Images were acquired as Z-stacks using LSCM-A and are presented with orthogonal views. Scale bars, 5 μm. **(E)** Cartoon depiction corresponding to the microscopy image in panel **(D)**. In *T. gondii* tachyzoites, the CENH3-3HA signal within each nucleus displays a discrete staining pattern. **(F)** HCT-8 cells were infected with excysted *C. parvum* sporozoites for 2 h, then washed twice to remove extracellular parasites. Cultures were fixed at 30 min increments between 6-9 hpi to identify the first (trophozoites with one nucleus and two centrin spots (1N2C)), second (early meronts with 2 nuclei, each with two centrin spots (2N2C)), and third (mid-stage meronts with 4 nuclei and 2 centrin spots (4N2C)) mitotic divisions during the first round of merogony by widefield microscopy. Cells were stained with a rabbit anti-Centrin-1 primary antibody, followed by an Alexa Fluor 568-conjugated goat anti-rabbit IgG secondary antibody and Hoechst. Scale bars, 1 μm. See also S3 Fig. **(G)** Cultures were infected with CENH3-3HA *C. parvum* sporozoites for 2 h, washed, and fixed at 6.5 hpi to capture the first round of mitosis. Cultures were stained with rat anti-HA, rabbit anti-centrin-1, followed by secondary antibodies Alexa Fluor 488 goat anti-rat IgG, Alexa Fluor 568 goat anti-rabbit IgG, and lastly Hoechst. Parasites undergoing mitosis were identified by having two centrin-1 points per nucleus (2N2C). Images were acquired as Z-stacks using LSCM-A and are presented with orthogonal views. Scale bars, 1 μm. Data represent 2-3 biological replicates, each with 3-5 technical replicates.

### Distribution of centrosomes in *C. parvum*

A vital step in mitosis is centrosome duplication, and previous studies in apicomplexans have shown that it can be traced using antibodies that recognize the centrin 1 protein [27,47]. A single point adjacent to the nucleus occurs in non-replicating cells, while two centrin 1 positive points per nucleus indicates the parasite is undergoing replication [27,47]. In *Plasmodium*, during schizogony, asynchronous duplications of centriolar plaques, centrosomes, have been observed across multiple nuclei within a single cytoplasm. In this study we sought to identify analogous centrosomes in *C. parvum* [28]*.* To identify parasites undergoing early mitosis, we infected HCT-8 cells with excysted wild type *C. parvum* sporozoites, washed off uninvaded sporozoites at 2 h, fixed the cultures at various timepoints between 6–9 hpi, and stained with Hoechst and an anti-centrin-1 antibody. Initially, trophozoites contained a single centrin-1 focus associated with the single nucleus (S3A Fig). However, in other examples of trophozoites, we also observed that each nucleus consistently exhibited two centrin-1 positive points (Fig 2F). Furthermore, by visualizing the number of nuclei based on Hoechst staining, and centrin-1 to monitor dividing centrosomes, we observed that all nuclei found in a common cytoplasm shared the same nuclear to centrin-1 staining pattern. For example, when 2N or 4N meronts were observed to have duplicated the centriole as shown by centrin-1 staining (designated as 2N2C or 4N2C patterns), the duplicated pattern was consistently observed for all nuclei within that cell (Fig 2F, S3 Fig). In no instance did we observe an odd number of centrin spots relative to nuclei- for example 2N vacuoles were never observed to have a collective 3C, only 2 or 4 (S3 Fig). To determine whether the diffuse centromere staining pattern persisted during active mitosis in *C. parvum*, we infected cultures with CENH3-3HA parasites and repeated the experiment previously described. We found that nuclei undergoing mitosis (2N2C) also displayed a diffuse CENH3-3HA staining pattern by LSCM-A (Fig 2G). We also attempted to localize CENH3 using ultrastructure expansion or immuno-EM staining methods. Despite extensive troubleshooting, we could not identify positive signals in N- or C-terminally tagged CENH3-3HA lines, or in a separate line expressing a spaghetti monster (sm) HA tag (i.e., CENH3-smHA), by either method, which may be due to the low abundance of CENH3 or difficulty preserving the epitope during these procedures.

### The telomere (G-strand binding protein, GBP) staining pattern is apical in *C. parvum*

Previous studies in *Plasmodium* have found that the telomeres are consistently found on the opposite pole to the centromeres [48–50]. Since the centromeres were located toward the apical end of the nuclei in meronts, we expected that *C. parvum* telomeres would be more basal. To examine the distribution of telomeres in *C. parvum*, we utilized a surrogate protein marker. G-quadruplex structures readily form at repetitive telomeric sequences and require G-quadruplex binding proteins (GBPs) for stabilization (Fig 3A). A previous study identified *cgd1_3530* as the GBP in *C. parvum* and determined the specific sequence necessary for *Cp*GBP binding (GGTTTA) [51]. Using AlphaFold and ChimeraX, we modeled the G-quadruplex structure formed by this repetitive sequence and visualized how the *Cp*GBP protein interacts with and stabilizes it. (Fig 3B). To identify the location of *C. parvum* telomeres relative to the centromeres, we engineered transgenic parasites to express a 3HA tag at the gene encoding the C-terminus of the G-quadruplex binding protein (referred to as GBP-3HA) (Fig 3C, S2A-B Fig, S1 Table). LSCM-A microscopy on infected HCT-8 monolayers (6.5 hpi) revealed that the telomeres were located toward the apical side of the nucleus (Fig 3D), rather than at the opposite pole as expected.

**Fig 3:**
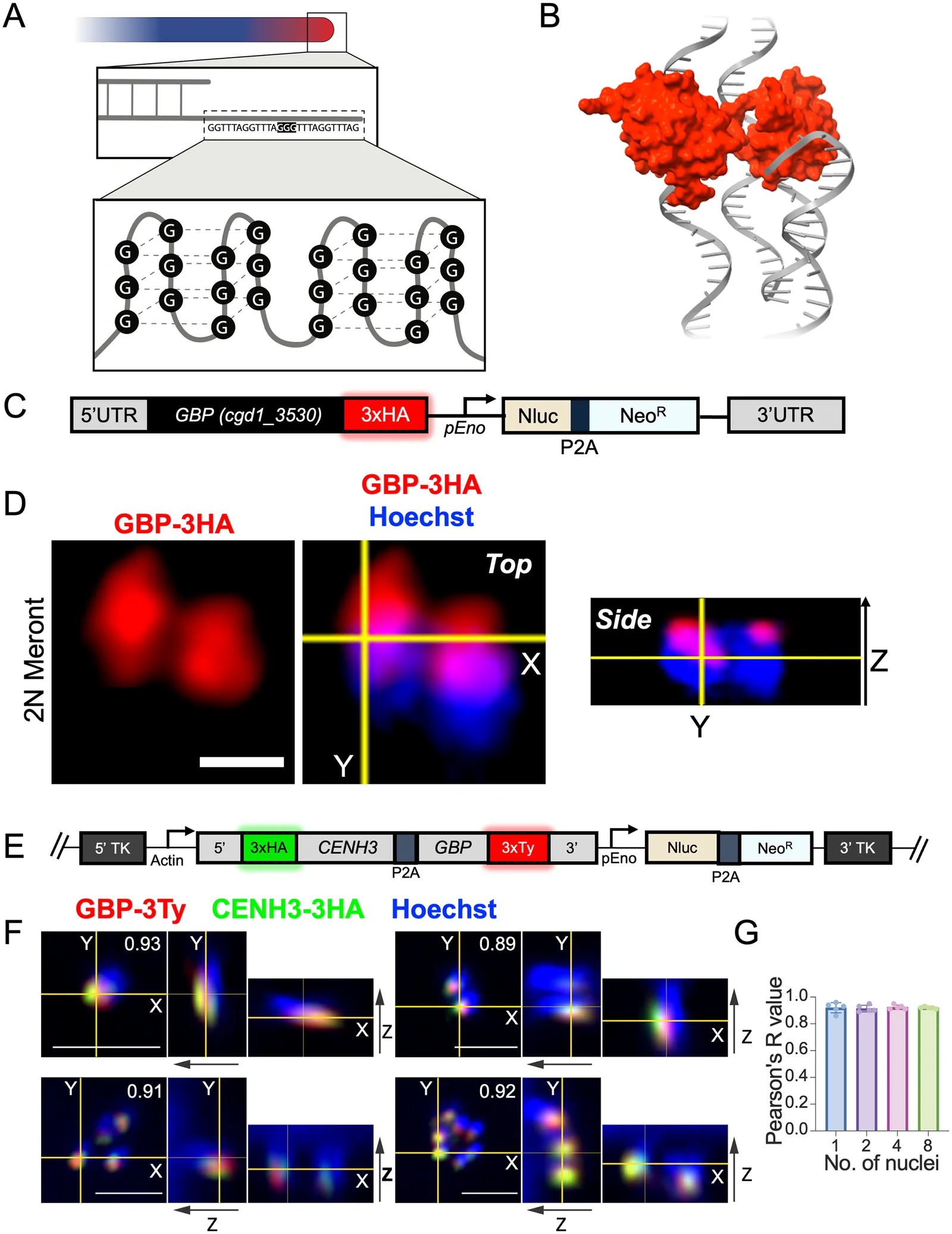
The telomere (G-strand binding protein, GBP) staining pattern is apical in *C. parvum* and overlaps with the centromere (CENH3) staining pattern. **(A)** The 3’ overhang single-stranded DNA at the end of telomeres contains repetitive sequences (GGTTTA)_n_ in *C. parvum* that form G-quadruplex structures. **(B)** The G-quadruplex binding protein (GBP) is essential for forming and stabilizing these structures. The sequence of *Cp*GBP and its binding motif has been previously determined and the protein structure is modeled by Alphafold and ChimeraX [51]. **(C)** Diagram of the targeting construct designed to add a 3HA tag and Nluc-P2A-Neo^R^ cassette to the C-terminus of the G-quadruplex binding proteins gene *(*GBP*, cgd1_3530)* **(D)** HCT-8 cells were infected with GBP-3HA parasites, fixed at 6.5 hpi to capture early meronts (2 nuclei), and stained with rat anti-HA, followed by the secondary antibody Alexa Fluor 488 goat anti-rat IgG. Hoechst was used for nuclear staining. Images were acquired as Z-stacks using LSCM-A and are presented with orthogonal views and the scale bar is 1 μm. **(E)** Diagram of the targeting construct designed to replace the endogenous *tk* locus with a second copy of CENH3 N-terminally tagged with 3HA and GBP with a C-terminal 3xTy tag separated with a P2A split peptide. **(F)** Immunofluorescence staining of transgenic 3HA-CENH3-GBP-3Ty parasites. HCT-8 cells were infected with transgenic oocysts, fixed at 9.5 hpi to capture all asexual stages during the first round of merogony, and stained with rat anti-HA and mouse anti-Ty, followed by a secondary antibody stain containing Alexa Fluor 488 goat anti-rat IgG and Alexa Fluor 568 goat-anti mouse. Hoechst was used for nuclear staining. Images were acquired as Z-stacks using LSCM-A and are shown as square panels, with corresponding orthogonal views shown as rectangular panels. Black arrows indicate the Z-axis. The numbers in the upper right corner of the merged images indicate the conventional Pearson correlation coefficient calculated from pixel-intensity correlations, reflecting the degree of colocalization between CENH3 and GBP signals. Scale bars, 2 μm. **(G)** Pearson correlation coefficients calculated from five representative images of each asexual stage. Representative images are shown in S4 Fig. Data represent 2-3 biological replicates, each with 3-5 technical replicates.

### The telomere (GBP) and centromere (CENH3) staining patterns overlap in *C. parvum*

To confirm that *C. parvum* centromeres and telomeres had overlapping nuclear locations, we generated an additional line of transgenic parasites that replaced the dispensable thymidine kinase (*tk*) gene with a second copy of an N-terminally 3HA tagged CENH3 and GBP C-terminally tagged with 3Ty via CRISPR/Cas9, with an intervening P2A split peptide to facilitate co-expression of separate proteins (referred to as 3HA-CENH3-GBP-Ty) (Fig 3E, S1C-D Fig, S1 Table). HCT-8 cultures were infected with 3HA-CENH3-GBP-Ty parasites, fixed at 9.5 hpi, and examined by LSCM-A microscopy after staining. We observed that 3HA-CENH3 and GBP-3Ty signals were both more concentrated in the apical region of the nucleus, as defined by Hoechst staining. This overlap was evident by fluorescence microscopy and further supported quantitatively using the Coloc2 plugin in FIJI/ImageJ, which yielded an average Pearson correlation coefficient of ~0.90 (Fig 3F,G, S4 Fig).

### Detection of mitotically active DNA, microtubules, and the nuclear pore proteins in *C. parvum*

The phosphorylation of histone H3 (specifically at serine 10 and serine 28) is a highly conserved mechanism for initiating chromosome condensation in preparation for mitosis [52]. Antibodies recognizing the phosphorylated version of H3 at serine 10 are commonly used in other systems to determine which cells are currently undergoing mitosis from late interphase through telophase [53]. Even though apicomplexan parasites are known not to condense their chromosomes, *C. parvum* meronts frequently stained positive for the phosphorylated histone H3 (PH3) antibody (Fig 4A), suggesting they are in the process of mitosis. Previous studies have characterized the distribution of nuclear pores in *Plasmodium falciparum* using a rabbit polyclonal antibody to the protein NUP116 [54]. We rationalized that the epitope recognized by this antibody might also be conserved in *C. parvum*, despite their evolutionary distance. We visualized nuclear pores in *C. parvum* using the rabbit anti- NUP116 antibody and found that it reliably stained the parasite nucleus in a punctate pattern that was concentrated primarily peripherally in the nucleus (Fig 4B). Although we have not verified the identity of this cross-reaction, it presumably represents a component of nuclear pores that will be used for future studies on nuclear organization in *C. parvum.*

**Fig 4:**
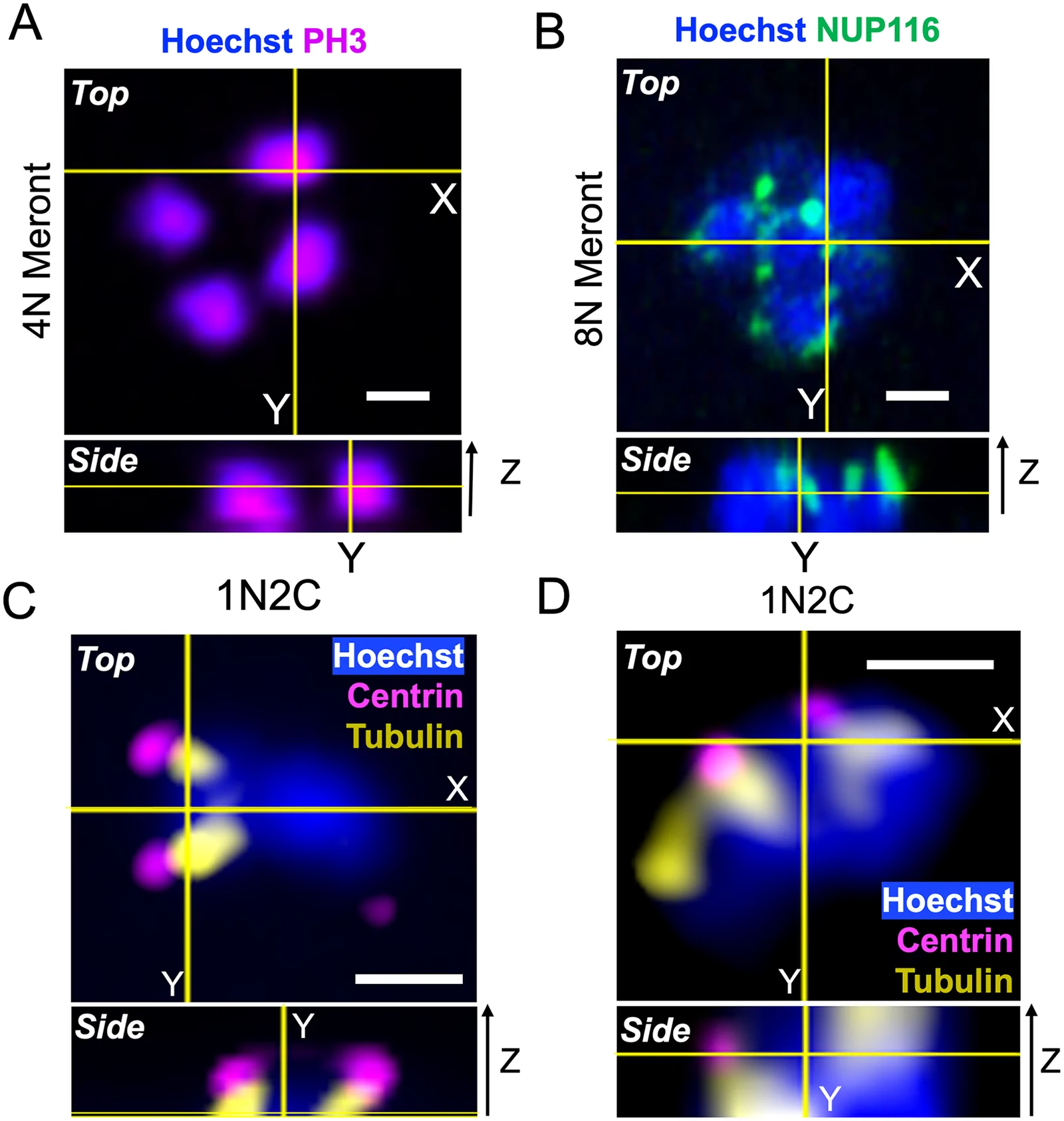
Mitotically active DNA, microtubules, and the nuclear pore proteins as visualized by antibody staining in *C. parvum.* **(A)** HCT-8 cells were infected with wild type parasites, fixed at 18 hpi, and stained with rabbit anti-phosphorylated Histone H3 (PH3) followed by an Alexa Fluor 568 goat anti-rabbit IgG antibody. DNA was stained with Hoechst and a mid-stage meront with 4 nuclei is shown. **(B)** Parasites were prepared identically to panel A, but were stained with a rabbit polyclonal antibody that recognizes a subunit of the nuclear pore in *Plasmodium falciparum* (NUP116) followed by an Alexa Fluor 568 goat anti-rabbit IgG antibody and Hoechst. A late-stage meront with 8 nuclei is shown. **(C, D)** HCT-8 cells were infected with wild-type parasites, fixed at 6.5 hpi to capture early meronts (2 nuclei), and stained with rabbit anti-centrin-1 and mouse anti-tubulin, followed by the secondary antibodies Alexa Fluor 488 goat anti-mouse IgG, Alexa Fluor 568 goat anti-rabbit IgG, and Hoechst. The nucleus in the first meront (C) may be an example of early mitosis, whereas the second meront (D) may be an example of a later phase of the mitotic process. Images were acquired as Z-stacks using LSCM-A and are presented with orthogonal views. Scale bars, 1 μm. Data represent two biological replicates, each with 2-5 technical replicates.

Among apicomplexan parasites, mitosis has primarily been studied in *T. gondii* and *P. falciparum* [16,17,27,55]. These prior studies demonstrate that once centrosomes duplicate, and the nuclear envelope forms a tunnel-like structure, organizing spindle microtubules within it, spindle microtubules then attach at the centromere and mediate chromosome movement [56]. We also attempted to visualize this process in *C. parvum* by staining with antibodies that recognize the centrin-1 and alpha tubulin (Fig 4C-D, S5A-B Fig). We examined cells at 6–7 hpi to capture early stages of mitosis. We frequently observed two conformations of centrin-1 tubulin structures that may represent early (Figs 4C, S5A) and late mitotic phases (Figs 4D, S5B). In Fig 4C, the duplicated centrin signal shows a single discrete spindle (microtubule signal) overlying what appears to be a single nucleus, as evident by size and shape (1N2C). In other examples the centrin spots have moved further away, and the spindles elongated (Y-shaped) (Figs 4D, S5B). Notably, the nucleus in this image is much larger and diffuse, consistent with it being at a later stage (labeled 1N2C although the nucleus might be diploid). Additionally, the spindle profiles in these images do not connect at a central midline. Similar diffuse microtubule staining associated with centrin-1 positive centrioles was previously reported by a study examining the role of gamma tubulin during nuclear division of *C. parvum* meronts [57]. Together, these images suggest that during nuclear division microtubules create an incomplete hemi-spindle in *C. parvum*, like what has been described in *Plasmodium* [58].

### Composition of centromeres in *C. parvum* and *T. gondii*

Centromeres are defined by the deposition of CENH3-containing nucleosomes [27,59]. Given the diffuse staining of CENH3, we wanted to further characterize the centromere by identifying DNA sites bound by this protein. Therefore, we adapted protocols for CUT&RUN to identify centromeres through CENH3 binding, based on capture with an anti-HA antibody in *C. parvum* [43]. To ensure sufficient HA binding, we developed a CENH3 line tagged with spaghetti monster (referred to as CENH3-smHA) that contains 10 HA epitope repeats for these experiments and validated identical staining patterns to CENH3-3HA *C. parvum* (S2A-C Fig, S1 Table). To validate our experimental strategy, we first applied CUT&RUN to *T. gondii*, whose centromeres have previously been defined by ChIP-seq [27]. Our CUT&RUN data closely matched earlier findings, with CENH3 enrichment observed at discrete chromosomal loci corresponding to the previously described centromeres (Fig 5A, S6A-C Fig). We also detected the positive correlation of CENH3 with H3K9me3 and inverse correlation with H3K4me3 reported in earlier ChIP-chip analyses (Fig 5A, S6A-B Fig). Based on these signals, we implemented a stepwise strategy to identify centromeric regions. Valleys in H3K4me3, consisting of 20 kb windows, sized to match reported centromere lengths (16 kb ± 3.4 kb) [27], combined with co-enrichment of H3K9me3 with CENH3-smHA were used as criteria for identifying centromere regions (S6D Fig). Applying these rules, we identified a single centromere in each of the 13 chromosomes of *T. gondii* (S6E Fig), with Chr VIIb identified as part of Chr VIII [60] (S6C Fig).

**Fig 5:**
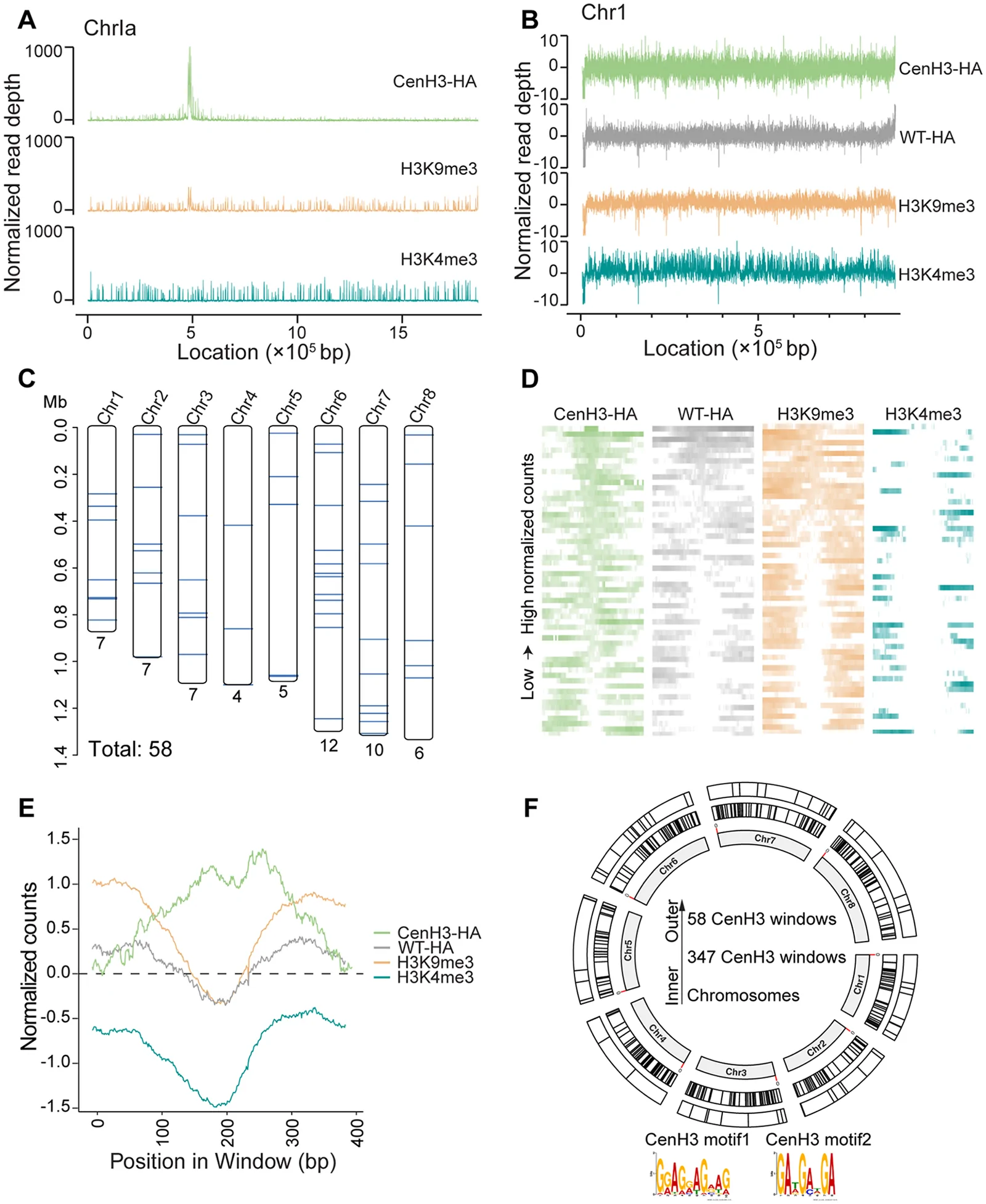
Genome-wide distribution of CENH3 in *Toxoplasma gondii* and *Cryptosporidium parvum.* **(A)** Genome browser view of *T. gondii* chromosome Ia showing CUT&RUN signals for CENH3-HA (HA antibody), H3K9me3, and H3K4me3. CENH3 signal enrichment positively correlates with H3K9me3 and inversely with H3K4me3. Peaks of CENH3 enrichment are indicated (red arrows). **(B)** Genome browser view of *C. parvum* chromosome I, showing CUT&RUN signals for CENH3-HA *C. parvum* captured with HA antibody and wild type *C. parvum* captured with HA antibody (referred to as WT-HA), H3K9me3, and H3K4me3 (captured with antibodies). **(C)** Chromosomal distribution of the 58 CENH3-enriched windows in intergenic regions of *C. parvum* chromosomes (Chr#). Numbers below each chromosome indicate the number of windows. **(D)** Heatmaps of CUT&RUN signals for the groups shown in (B) within a 400 bp window around all 58 CENH3-enriched windows. Each row represents an individual centromeric site, ranked by signal intensity. **(E)** Average signal profiles for CENH3-HA (green), WT-HA (gray), H3K9me3 (orange), and H3K4me3 (teal) across the 400 bp window around CENH3 sites. **(F)** Circos plot of CENH3-associated motifs. From inner to outer rings: chromosomes, locations of 347 CENH3-enriched windows found in coding regions (black), and locations of 58 CENH3-enriched windows found in intergenic regions (black). Representative sequence logos for motif 1 and motif 2 are shown.

We next applied CUT&RUN to *C. parvum*. Initial experiments using a 120-minute MNase digestion failed to generate usable data, as both sequencing depth and genome coverage were low compared to negative controls (S1 Data). Since CENH3 nucleosomes can be hypersensitive to MNase digestion, as reported previously in *Caenorhabditis elegans* (*C. elegans)* [59], we tested digestion times of 5, 30, and 60 min. The 30 min digestion yielded the best coverage and sequencing depth, and these conditions were used for following analyses (S1 Data, S7A Fig). Notably, sequencing depth of the *C. parvum* samples was more variable and, in some cases, lower than that of *T. gondii*. This difference primarily reflects technical challenges associated with generating transgenic *C. parvum* parasites. To ensure data quality and comparability, we included only samples with high genome coverage (>80%) in downstream analyses. Unlike *T. gondii*, read mapping profiles for CENH3 in *C. parvum* showed signals broadly distributed along chromosomes (Fig 5B). To locate centromeric regions in *C. parvum*, we first attempted the *T. gondii* stepwise strategy, but no valid windows were detected. As the CENH3 distribution resembled that of the holocentric nematode *C. elegans*, we adjusted thresholds accordingly by restricting the peaks analysis to 400 bp windows (S7B Fig). We identified 423 CENH3-enriched regions distributed across all chromosomes. Heatmaps and aggregate signal profiles showed that these windows displayed features of CENH3 enrichment correlated with H3K9me3 deposition and H3K4me3 depletion, suggesting that they represent centromere-associated chromatin (S7C-D Fig). Importantly, these signals were largely absent in wild type parasites stained with HA antibody as a control, supporting their authenticity (S7C-D Fig). To further refine localization, we excluded windows overlapping with WT-HA signals, reducing the dataset to 347 high-confidence centromeric windows (S7B,E Fig). Taken together, these results indicate that *C. parvum* possesses many small CENH3 binding sites dispersed across eight chromosomes, in contrast to the monocentric nature of *T. gondii*.

We further investigated characteristics of *C. parvum* centromeres. In contrast to *T. gondii*, where centromeres are typically located within large intergenic regions [27], no extended intergenic regions were observed in *C. parvum* due to the tight compaction of the genome. Surprisingly, the majority of *C. parvum* CENH3-associated windows were located within gene coding regions rather than being restricted to intergenic regions (S2 Data). To evaluate evolutionary constraints on CENH3-associated regions, we performed nucleotide diversity (Pi) analyses on the DNA sequences within these regions and dN/dS analyses on the genes overlapping these windows. We compared previously generated genomic sequences from numerous *C. parvum* IIa and IId isolates, which show high genomic similarity. Most CENH3-associated windows (~82%) showed reduced Pi values compared with genome-wide 400-bp sliding windows, and only a single gene within the windows exhibited an elevated dN/dS ratio relative to the genome-wide orthologous gene set (S7F,G Fig). These results indicate that CENH3-associated regions are highly conserved and under strong purifying selection. To determine whether centromeric regions were also present within intergenic regions of *C. parvum*, we applied a stepwise identification strategy using a slightly relaxed read depth threshold (S7F Fig). We identified 58 centromeric regions distributed within intergenic regions across all chromosomes (Fig 5C, S3 Data). Heatmaps and aggregate signal profiles for CENH3 as well as chromatin marks also supported that they represent centromere-associated chromatin (Fig. 5D,E). These results suggest that CENH3 binding in *C. parvum* is broadly distributed throughout the chromosomes, including both within coding regions and intergenic regions. To assess sequence properties of *C. parvum* centromeric windows, motif analysis of *C. parvum* centromeric windows was performed using MEME, revealing two GA-rich motifs enriched at CENH3-associated sites (Fig 5F). Notably, these GA repeats are also found in other regions of the genome that were not identified in the CENH3 capture studies.

## Discussion

Despite rapid asexual proliferation being required for parasite expansion, the molecular mechanisms that underlie *C. parvum* mitosis have remained unexplored. To investigate this process, we examined nuclear division using markers to track multiple rounds of mitosis during merogony*.* Nuclear division in *C. parvum* can be visualized by staining with phospho-H3 and centrin-1, which provide markers for mitotically active chromatin and duplicating centrosomes, respectively. Surprisingly, both CENH3, a marker for centromeres, and GBP, a marker for telomeres, were diffusely localized across the apical half of the nucleus. Moreover, these diffuse apical patterns persisted throughout mitosis, as shown by visualizing nuclear division stages with centrin-1 and microtubules. To explore the molecular nature of centromeres, we performed CUT&RUN sequencing of transgenic parasites expressing epitope-tagged CENH3. Although discrete monocentric peaks were detected in the related parasite *T. gondii*, CENH3 binding sites were diffusely scattered across ~ > 400 sites in the *C. parvum* genome. Overall, these findings indicate that *C. parvum* has evolved an alternative chromosome structure compared to other apicomplexan parasites, differences that may have significant functional consequences for chromosome stability and meiotic recombination.

Following entry of sporozoites (or merozoites) into the host cell, the parasite undergoes merogony, consisting of 3 rounds of nuclear division prior to cytokinesis to generate infectious merozoites. By visualizing the number of nuclei based on Hoechst staining and centrin-1 to monitor dividing centrosomes, we observed that all nuclei found in a common cytoplasm shared the same centrin-1 staining pattern (a single centrin-1 spot when not dividing and a duplicated centrin-1 spot before nuclear division). This pattern suggests that nuclei within a single meront may divide synchronously, although further studies using synchronized cells and live cell imaging would be needed to substantiate this hypothesis. This pattern differs from *Plasmodium* schizogony, where nuclei are observed at different mitotic stages within a single schizont [28]. In *Plasmodium*, nuclear pores are important for division as they facilitate the transport of mitotic molecules through the intact nuclear envelope and associate with the centriolar plaques [31,61]. Although we did not examine the dynamics of nuclear pores during division, they appear as peripheral puncta in mature meronts and may serve a similar function in *C. parvum*.

Staining with alpha-tubulin allowed us to visualize *C. parvum* microtubules during mitosis, particularly at early stages where microtubules form a cluster that was often branched or incomplete at the center. Similar profiles have been described previously in *C. parvum* [57] and they are reminiscent of the hemi-spindles described in other apicomplexans [58]. The pattern of microtubules described here in dividing cells is quite different from the stable, elongated microtubules that emanate from the apical pole of sporozoites and merozoites [57]. Such dramatic re-organization of microtubules between non-dividing and dividing cells is not unusual for eukaryotic cells, although the dynamics and regulation of this process have not been examined extensively in *Cryptosporidium*. Future studies using fluorescently tagged proteins and live cell imaging, combined with synchronous cell division would be important to further elucidate the dynamics of cytoskeletal changes during mitosis in *C. parvum.*

We identified three histone H3 variants in *C. parvum* using reciprocal BLAST and phylogenetic analyses including two conventional variants and one more divergent form annotated as CENH3. The two conventional variants were arbitrarily named H3.1 and H3.2, designations that were not meant to imply functionality. Normally H3.1 and H3.2 in mammals are replication coupled and contain key sequence motifs such as QSSAV [62]. This motif is found in *cgd4_3220*, which would suggest it is replication coupled. In contrast, *cgd3_2540* contains the sequence QSQAV, making it more similar to H3.3 variants previously described in *T. gondii* and *P. falciparum* [63]. To what extent these subtle sequence differences might affect function would require further testing. All three variants showed a diffuse nuclear staining pattern. Although this pattern is expected for conventional H3 variants, it was surprising for CENH3–3HA where the diffuse pattern sharply contrasts with the punctate pattern observed in other apicomplexan parasites [27,29,34]. We also confirmed that the diffuse CENH3 staining pattern did not deviate when the method of tagging was altered (e.g. smHA), when the location of the tag was swapped (N-terminally tagged, e.g., 3HA-CENH3-GBP-3Ty), or when a tagged second copy of the gene was inserted into a neutral locus of the genome (3HA-CENH3-GBP-3Ty). We also developed epitope-tagged lines of telomere-binding protein GBP, similar to studies in *Eimeria* and *Plasmodium* [64,65]. Unlike in other apicomplexans, GBP was also localized to the apical half of the nucleus in *C. parvum*, overlapping closely with CENH3 [18–20,48]. We further confirmed that the telomeres and centromeres occupied a similar nuclear location using a parasite strain in which CENH3 and GBP proteins were simultaneously expressed as tagged second copies. This pattern contrasts sharply with other apicomplexans where telomeres are localized to the opposite pole of the nucleus from centromeres [48–50]. We further confirmed that in *C. parvum,* the diffuse centromere staining pattern does not change throughout mitosis, which further contrasts with the highly focal nature of centromeres in *T. gondii* and *P. falciparum* [26,27].

Given the highly unusual staining patterns of CENH3 in *C. parvum*, we decided to investigate CENH3 binding sites on DNA using CUT&RUN sequencing. For comparison, we also performed CUT&RUN studies on CENH3 in the related parasite *T. gondii* and compared them to previous studies that used ChIPSeq to define centromeres [27]. In *T. gondii*, CUT&RUN sequencing identified highly similar CENH3 binding sites to those reported previously by ChIPSeq [27] with a single predominant peak on each chromosome, including newly defined peaks on chromosome IV, that had not been identified previously. Centromeres in *T. gondii* consist of gene-poor regions of ~15 kb that are not defined by any particular sequence or repeats but instead are positively correlated with H3K9me3 and inversely correlated with H3K4me3, typical chromatin patterns expected for centromeric regions [18,27]. Under similar conditions, CUT&RUN experiments for CENH3 binding sites in *C. parvum* failed to identify similar singular dominant peaks on each chromosome. Instead, CENH3 binding sites were hypersensitive to MNase digestion, with the signal being the highest at a 30- min incubation time, similar to that observed in ChIP-seq studies of the holocentric nematode *Caenorhabditis elegans* (*C. elegans*) [59]. Under optimal digestive conditions, we found that the CENH3 signal in *C. parvum* was broadly distributed along chromosomes, with ~ 350 distinct sites in coding regions and another ~ 60 sites within intergenic regions scattered across 8 chromosomes. Interestingly, like *T. gondii*, *C. parvum* CENH3 peaks also correlated with H3K9me3 signal and were found in areas lacking H3K4me3, consistent with a centromere-associated chromatin pattern. The distribution of CENH3 binding sites in *C. parvum* is similar to that described in *C. elegans* as polycentric, implying that CENH3 binds to distinct sites rather than diffusely across the entire chromosome [59].

Collectively, our findings are consistent with *C. parvum* containing holocentric centromeres based on the following criteria: Firstly, the staining pattern of CENH3 diffusely stains the apical half of the nucleus under nondividing conditions, in contrast to the focal staining of CENH3 in *T. gondii* [18,27] and *P. falciparum* [26]. Secondly, the diffuse pattern of CENH3 staining remains throughout mitosis, suggesting centromeres do not coalesce into a single regional center. Thirdly, CENH3 binding sites as defined by CUT&RUN resemble the dispersed pattern of low-intensity peaks seen in *C. elegans*, which has well-characterized holocentric chromosomes. Although we interpret this diffuse distribution as consistent with holocentric centromeres, we acknowledge that an alternative explanation, such as asynchronous chromatid separation or cohesion fatigue, could also contribute to the observed pattern. However, current imaging resolution does not allow simultaneous visualization of all microtubules and all CENH3-associated kinetochore sites, so we cannot fully distinguish between these possibilities. Therefore, we present our interpretation cautiously while noting that the number of predicted CENH3 loci substantially exceeds the 8 (1N) regional centromeres expected for a monocentric organization. Finally, the CENH3 binding sites show evidence of simple GA nucleotide repeats, a feature also seen in *C. elegan*s [59]. In both cases, the GA repeats are also found in other regions of the respective genomes, so this feature alone is not sufficient to mediate CENH3 binding. One unusual feature of the CENH3 binding sites in *C. parvum* is that they extend into coding regions, which is not an expected feature of centromeres. Interestingly, these regions were under strong purifying selection at the intraspecies level. The dispersed polycentric pattern of CENH3 binding sites in *C. elegans* is also unusual in that these regions are concentrated in High Occupancy Targets (HOT) sites for permissive binding of transcription factors [59]. In *C. elegans*, the CENH3 and HOT sites appear to serve a dual purpose: in replicating cells they bind CENH3, while they recruit transcription factors when CENH3 is lost during exit from the cell cycle in differentiated muscle cells [59]. By analogy, transcriptional silencing during mitosis in *C. parvum* may allow CENH3 binding in coding regions to facilitate chromatid segregation, with displacement during the rest of the cell cycle when transcription is active. Alternatively, the CENH3 binding sites in intergenic regions may function as the primary centromere-localizing signal for formation of kinetochores.

Holocentric chromosomes have been documented in many species, primarily through direct cytological observation, yet their functional implications and evolutionary origins remain poorly understood [37,59,66,67]. Recent comparative and modeling work in fungi shows that complete transitions between centromere ‘types’ (point/regional/holocentric) occur progressively and do not fix by drift alone; rather, selection is needed to drive and stabilize new architectures while maintaining compatibility with the relatively conserved kinetochore–spindle interface [68]. In this light, the many small CENH3-binding sites in *C. parvum* fits a model of progressive centromere remodeling along an evolutionary continuum, rather than representing an abrupt departure from other apicomplexan centromere types.

The organization we describe has clear functional implications, as the presence of hundreds of small CENH3-positive sites suggests that *C. parvum* chromosomes may rely on numerous ‘micro-kinetochores’ distributed along their length. By spreading spindle attachment across many small units rather than concentrating mechanical force at a single centromere, the parasite could effectively diffuse the load generated during chromosome segregation. Such a system may be particularly advantageous for *C. parvum*, which performs closed mitosis with uncondensed chromatin and must execute multiple rounds of synchronous division during rapid merogony.

Current evidence suggests that holocentric chromosomes have evolved at least 13 times convergently across distantly related taxa in both the plant and animal kingdoms [36,37,67]. Holocentricity has not previously been described in protists, and hence it likely evolved convergently in *C*. *parvum*. However, we note that the directionality of centromere evolution within Apicomplexa remains unresolved. Because centromere organization has not been characterized in closely related outgroups such as gregarines or chromerids, it is equally plausible that the common ancestor of Apicomplexa possessed holocentric chromosomes and that monocentric organization arose secondarily in coccidia and hematozoa. Thus, the holocentric architecture in *C. parvum* may represent either an independently derived state or the retention of an ancestral condition. It is also possible that holocentricity is more widespread among protists, but that it has been overlooked due to their small size and failure to condense chromosomes during division.

One proposed advantage of holocentricity is its capacity to stabilize chromosomal fragments during mitosis, thereby mitigating genetic loss. This trait may confer a selective benefit to organisms frequently exposed to elevated levels of DNA damage due to environmental stressors or lifestyle factors [36,66]. Thus, holocentricity may impart genome stability under stress encountered in the gastrointestinal system where the parasite is exposed to toxic bacterial metabolites [69] and host immune responses [70]. Despite this potential advantage, holocentricity also presents a major challenge for coordinating chromatid segregation following crossover during meiosis I. Well-characterized holocentric species have evolved mechanisms to circumvent this issue, such as inverted meiosis or, in the case of *C. elegans*, the deployment of kinetochore proteins that localize to discrete chromosomal regions, effectively mimicking monocentric behavior [71]. Whether *C. parvum* uses a similar mechanism as *C. elegans* to localize kinetochore proteins to discrete sites on the chromosome is uncertain, but previous work from our laboratory examining crossover rates during *C. parvum* meiosis revealed a high frequency of crossover events and failed to find evidence for inverted meiosis, a process used by some holocentric organisms [72]. In contrast, interspecies crosses between *C. tyzzeri* and *C. parvum* revealed that crossovers are strongly suppressed and most chromosomes are inherited uniparentally without crossovers [73]. Although we have not interrogated the nature of CENH3 binding sites in *C. tyzzeri*, differences in the recognition sequences or pattern of centromere distribution (i.e., monocentric vs. holocentric) could result in suppression of hybrids due to mitotic or meiotic interference.

### Limitations and future studies

Examination of the nuclear organization of *C. parvum* throughout the cell cycle revealed a persistently diffuse centromere staining pattern, suggesting a holocentric chromosome structure. During division, such multiple centromeric sites dispersed along the chromosome are expected to bind centrosomes and thereby connect to microtubules for chromosome separation. Although we visualized events consistent with this process in an artificial timeline, future studies using fluorescent reporters could potentially provide more precise information on the formation of centrosomes, coordinated assembly of microtubules, and nuclear division in *C. parvum*. However, obtaining direct cytological data in support of holocentric centromeres and their behavior during nuclear division will be challenging in this system because chromosomes do not condense during division. Alternatively, it may be possible to explore the nature of centromeres by examining the distribution of other conserved centrosomes and kinetochore binding proteins during nuclear division. Such studies could shed further light on chromosome tethering in nuclear division and provide invaluable insights into unique evolutionary adaptations in *C. parvum*. Additionally, extending these studies to define how centromeres are organized in related species could provide insight into mechanisms of diversification that drive speciation.

## Materials and methods

### Animal studies and ethical approval

Animal studies were approved by the Institutional Animal Studies Committee (School of Medicine, Washington University in St. Louis). *Ifngr1*^-/-^ mice (003288; Jackson Laboratories), and NOD scid gamma mice (referred to as NSG) (005557; Jackson Laboratories) were bred in-house in a specific-pathogen-free animal facility on a 12:12 light-dark cycle. Male and female mice between 8 and 12 weeks of age were used for sex-matched groups used in crossing experiments. Mice were co-housed with siblings of the same sex throughout the experiments.

### Cell culture

HCT-8 cells (Human ileocecal adenocarcinoma, ATCC CCL-244) derived from a human ileocecal carcinoma were cultured in RPMI 1640 ATCC Modification medium supplemented with 10% fetal bovine serum at 37 °C in a 5% CO_2_ incubator. After trypsinization, 450,000 cells were plated on glass coverslips in 24-well plates and used for experiments. Cells were confirmed to be mycoplasma-free with the e-Myco plus Mycoplasma PCR detection kit.

**Immunofluorescence assays & microscopy.** Staining protocols have been previously described and all antibodies are listed in S2 Table [74]. Briefly, infected HCT-8 cultures were fixed with 4% formaldehyde 6–24 hpi, permeabilized with 0.1% Triton X-100, blocked with 1% bovine serum albumin (BSA), stained with a primary antibody mixture for 1 h followed by secondary antibodies for 1 h and Hoechst for 20 min. Primary antibodies were diluted for staining: anti-HA (rat, 1:500), anti-Aldolase (ALD, rabbit, 1:1000) centrin-1 (rabbit, 1:250), anti-Ty (mouse, 1:250), and anti-PH3 (rabbit, 1:500), NUP116 (rabbit, 1:100). All Alexa Fluor secondary antibodies were diluted at 1:1000 and Hoechst was diluted 1:2000. Coverslips were mounted onto microscope slides with Prolong Glass Antifade Mountant. Coverslips were visualized using a Zeiss LSM880 laser scanning confocal microscope equipped with a 63x, 1.4 N.A. Zeiss Plan Apochromat oil objective lens and Airy scan. Images were acquired with ZEN v2.1, v2.5 software and further analyzed using ImageJ. All staining experiments were performed independently 2–3 times, and at least 2–5 fields of view were imaged per coverslip.

**Expansion Microscopy.** Ultrastructure expansion microscopy (U-ExM) was performed as previously described [75]. HCT-8 cells were infected with the CENH3-3HA or CENH3-smHA parasites and fixed following the same procedures used for standard immunofluorescence assays or 16% methanol-free formaldehyde. Fixed samples were embedded overnight in a solution containing 1% acrylamide and 0.7% formaldehyde to anchor proteins and prevent crosslinking. The resulting gel was transferred into denaturation buffer and incubated at 95 °C. A second expansion gel was polymerized on ice using a monomer mixture composed of 19% sodium acrylate, 10% acrylamide, and 0.1% (1,2-Dihydroxyethylene) bisacrylamide, supplemented with 0.5% ammonium persulfate (APS) and 0.5% TEMED. Polymerized gels were denatured at 95 °C for 90 min in denaturation buffer (200 mM SDS, 200 mM NaCl, 50 mM Tris-Base, pH 9.0) and expanded overnight in ultrapure water. Expansion ratios were determined the following day by measuring gel diameters. Gels were then shrunk in PBS and stained with primary antibodies (rabbit anti-HA, rat anti-HA, or either rabbit anti-GFP antibodies at 1:200 and rat Pan-Cp, 1:500), followed by secondary antibodies (Alexa Fluor conjugates, 1:500) and Hoechst (1:500) diluted in freshly prepared 2% PBS/BSA for 3, 12, or 24 h for primary antibodies and 2 h for secondary antibodies at room temperature. After each staining step, gels were washed three times in PBS/0.1% Tween for 10 min. Stained gels were re-expanded overnight in ultrapure water before imaging. Images were acquired using a Zeiss LSM880 laser scanning confocal microscope equipped with a 63x, 1.4 N.A. Zeiss Plan-Apochromat oil objective and ZEN v2.1 or v2.5 software.

**Immuno-electron Microscopy.** Immuno-electron microscopy (EM) was performed as previously described using CENH3-3HA and CENH3-smHA parasites [76]. HCT-8 monolayers were grown in 6-well plates and reached confluence before infection with transgenic *C. parvum* oocysts. After a 20-h incubation, cultures were washed twice in DPBS and fixed for 1 h at 4 °C in freshly prepared fixative consisting of 4% paraformaldehyde and 0.05% glutaraldehyde in 100 mM PIPES supplemented with 0.5 mM MgCl_2_ (pH 7.2). Fixed samples were embedded in 10% gelatin and subsequently infiltrated overnight at 4 °C with a cryoprotectant solution of 2.3 M sucrose and 20% polyvinyl pyrrolidone prepared in PIPES/MgCl_2_. Blocks were trimmed, rapidly frozen in liquid nitrogen, and sectioned using a Leica Ultracut UCT7 cryo-ultramicrotome. Ultrathin sections (50 nm) were blocked for 30 min in a mixture of 5% fetal bovine serum and 5% normal goat serum, followed by incubation with rabbit anti-HA, rat anti-HA, or either of the rabbit anti-GFP antibodies (1:1000) for 1 h at room temperature. After rinsing in blocking buffer, sections were labeled for 1 h with 18 nm colloidal gold–conjugated goat anti-rabbit IgG (H + L) diluted 1:20. Labeled sections were then contrasted with 0.3% uranyl acetate in 2% methyl cellulose and imaged using a JEOL 1200 EX transmission electron microscope equipped with an AMT 8-megapixel digital camera and AMT Image Capture Engine v6.0.2. Control sections processed in parallel without primary antibody were included in all experiments.

### CRISPR/Cas9 plasmid construction

#### Plasmid construction.

Primers used to generate the following plasmids were ordered from IDT and all sequences are found in S3 Table. SnapGene (v7.1.1) and NEBuilder Assembly Tool (v2.9.0) were used to construct plasmid maps and design primers.

The CENH3-3HA tagging plasmid was generated using the previously described ABC-3HA tagging plasmid [76] by replacing the C-terminal and 3’UTR sequences with equivalent regions from the *Cp*CENH3 gene amplified from *C. parvum* cDNA to generate the tagging plasmid CENH3-3HA-Nluc-P2A-Neo^R^.

The 3HA-CENH3- GBP-3Ty tagging plasmid was created via a seven-way Gibson assembly combining a backbone generated from the TK*-*GFP-Nluc-P2A-Neo^R^-TK homology tagging plasmid previously generated in our lab [77] together with the following fragments: 1) The 5’ homology region of CENH3 (500 bp), the CENH3 CDS, a portion of the 3’ homology region of CENH3 (216 bp) were amplified from the CENH3-3HA tagging plasmid described above. 2) The 3HA-pLinker region was amplified from a TK-3HA-CENH3-Nluc-P2A-Neo^R^-TK plasmid 3) the GBP CDS was amplified from *C. parvum* genomic DNA, 4) the pLinker-3Ty sequence was amplified from a tagging plasmid previously generated in our lab [76], and 5) the P2A skip peptide sequence was generated as a gBlock Gene Fragment from Azenta. The U6:sgTK plasmid previously generated in our lab was utilized for all experiments with TK locus replacement repair plasmids [77].

The H3.1-3HA and H3.2-3HA tagging plasmids were generated via Gibson assembly using the 3HA-CENH3-GBP-3Ty plasmid to provide a backbone, the pLinker-3HA region was amplified from the CENH3-3HA tagging plasmid described above, and the CDS regions for H3.1 and H3.2 were amplified from *C. parvum* genomic DNA.

The GBP-3HA tagging plasmid was generated using the CENH3-3HA tagging plasmid described above for the backbone and replacing the C-terminal and 3’UTR sequences with the GBP CDS sequence amplified from *C. parvum* genomic DNA.

CENH3 and GBP CRISPR/Cas9 plasmids were created via Gibson assembly of the linearized backbone amplified from the TK CRISPR/Cas9 plasmid (Addgene 122852) and an oligonucleotide with the sgRNA specific for the CENH3 or GBP gene [77]. The Eukaryotic Pathogen CRISPR guide RNA/DNA Design tool (http://grna.ctegd.uga.edu) was used to identify CENH3 and GBP sgRNA sequences.

### Amplifying transgenic parasites in mice

The transfection and amplification of parasites in immunocompromised mice were conducted as previously described [77–80]. Briefly, 1x10^8^ freshly excysted sporozoites were electroporated with 100 μg of repair plasmid and 66 μg of CRISPR/Cas9 plasmid, then resuspended in 300 μL of cold DPBS. *Ifngr1*^-/-^ mice were first orally gavaged with an 8% (wt/vol) sodium bicarbonate solution to neutralize stomach acid, followed by the administration of the electroporated sporozoites. To facilitate the selection of transgenic parasites, paromomycin (16 g/L) was provided in the drinking water 24 h post-infection. Fecal pellets were collected at intervals post-infection for luciferase assays and diagnostic PCR analysis to verify parasite growth and proper gene insertion. Oocysts were isolated from fecal material using bleach treatment, and 20,000 oocysts per mouse were employed for a subsequent round of amplification in NSG mice.

**Luciferase assays for mouse experiments.** Fecal samples collected from infected mice were analyzed with the Promega Nano-Glo luciferase assay kit, as previously described [80]. The luciferase activity was measured using the BioTek Cytation 3 cell imaging multi-mode reader. The number of relative luminescence units per milligram of feces was obtained by dividing the average values from two technical replicates by the fecal sample weight.

**Quantifying oocyst shedding in mouse experiments.** DNA was extracted from fecal pellets collected from infected mice using the QIAamp PowerFecal DNA kit, following previously established methods [77,79,80]. Quantitative PCR (qPCR) analysis was conducted to determine the number of oocysts at each time point based on *C. parvum* glyceraldehyde-3-phosphate dehydrogenase (GAPDH) primers and a standard curve derived from a dilution series (S2 Table) [77]. Genomic DNA (gDNA) amounts were quantified using the QuantStudio 3 real-time PCR system.

### Confirmation of insertions in transgenic parasites by PCR

To confirm the correct integration of the homology repair template, extracted gDNA from murine fecal samples and wild type controls were used as the template. PCR amplification was performed using 1 µL of the extracted gDNA, Q5 Hot Start high-fidelity 2X master mix or PrimeSTAR GXL premix (TaKaRa), and primers at a final concentration of 500 nM on a Veriti 96-well thermal cycler. All PCR products were resolved on a 1% agarose gel containing GelRed (1:10,000) and visualized using the ChemiDoc MP imaging system.

### Oocyst purification from fecal material and storage

The *C. parvum* isolate AUCP -1 used for transfections was purified from fecal material collected from male Holstein calves at the University of Illinois at Urbana-Champaign [81]. All procedures involving the calves received approval from the Institutional Animal Care and Use Committee (IACUC). Transgenic oocysts were purified in-house from fecal material collected from NSG mice, using a saturated sodium chloride solution for oocyst flotation [82]. Post-purification, oocysts were stored at 4°C in phosphate-buffered saline (PBS) for up to three months following fecal collection.

### Structural predictions

The protein structure of the G-quadruplex binding protein (GBP, *cgd1_*3530) was predicted using Alphafold 3 (www.alphafoldserver.com). Using ChimeraX (https://www.cgl.ucsf.edu/chimerax/), we modeled how the G-quadruplex structure formed by the *Cp*GBP repetitive binding sequence (GGTTTA) [51] interacts and is stabilized by the CpGBP protein.

### Channel colocalization quantification

Co-localization analysis was performed using the Coloc2 plugin (FIJI/ImageJ), using default thresholding parameters. A 2D intensity histogram was generated as part of the Coloc2 output to visualize the pixel-wise distribution of intensities from both channels and a Pearson correlation coefficient was quantified.

### Parasite Preparation and Lysis for CUT&RUN experiments

The CENH3-3HA line of *T. gondii* [27] was obtained from the laboratory of Dr. Kami Kim at University of South Florida. *Tg*CENH3-3HA tachyzoites were propagated in human foreskin fibroblasts and harvested using established protocols [83]. *C. parvum* sporozoites (4x10^7^) and *T. gondii* tachyzoites (1x10^7^) were centrifuged at 2,500 RPM for 5 min, supernatants removed, and parasites were resuspended in 2 mL of cold parasite lysis buffer (10 mM HEPES pH 7.9, 1.5 mM MgCl2, 10 mM KCl, 0.65% NP40 (supplemented with IGEPAL) and 0.5 mM PMSF (supplemented with cOmplete EDTA free protease inhibitor cocktail and sterile distilled H_2_O), incubated for 10 min on ice, and centrifuged at 3,000 RPM for 10 min at 4°C (previously described in [84,85]).

### CUT&RUN processing, library preparation, quality control and sequencing

Following lysis treatment, the lysis buffer was removed, and the nuclei pellet was washed following the CUT&RUN Kit Version 4 protocol (EpiCypher, Inc, Catalog No. 14-1048). Isolated nuclei were incubated with either 1.0 μg of rat anti-HA antibody, 0.5 μg of rabbit anti-H3K4me3 positive control antibody, or 0.5 μg of mouse anti-IgG negative control antibody. Chromatin digestion and release steps were performed according to the EpiCypher protocol, and 5 ng of CUT&RUN enriched DNA was used for library preparation according to the CUT&RUN Library Prep Kit User Manual Version 1.5 (EpiCypher, Inc, Catalog No. 14-1001). The concentrations of CUT&RUN library samples were determined via Qubit and quality control were assessed with an Agilent 4200 TapeStation.

### CUT&RUN analysis and visualization

Multiple CUT&RUN runs were preformed to obtain biological replicates of each sample. DNA products were sequenced on an Illumina NovaSeq 6000 using 150-bp paired-ends at the GTAC sequencing core at Washington University in St. Louis. Sequence reads obtained from Illumina were trimmed for adapter sequences and poor sequence quality (phred score < 25 and trimmed length < 50) using fastp v0.23.4. The trimmed reads were mapped to the reference *C. parvum* IOWA-CDC genome using BWA-MEM v0.7.17 [86]. Duplicate reads were marked and removed in the bam files using the MarkDuplicates algorithm of picard v3.1.1 (https://broadinstitute.github.io/picard/) with REMOVE_DUPLICATES=true, CREATE_INDEX=true, and ASSUME_SORTED=true. Reads with mapping lengths less than 50 bp were further removed from the bam files using SAMtools v1.7 (http://samtools.sourceforge.net/). Genome coverage and sequence depth were estimated using the mpileup algorithm of SAMtools. The read depth of each locus was normalized by dividing by the total number of counts for all loci, then scaled by multiplying by the number of loci in the genome. Input counts were subtracted from negative control counts. Data was visualized by a R package ‘ggplot2’ (https://www.rdocumentation.org/packages/ggplot2). Motifs within centromeric windows were identified using MEME v5.5.8 (https://meme-suite.org/meme/tools/meme). Chromosomal distributions of intergenic regions, centromeric windows, and motifs were visualized with Circos plot generated in TBtools-II v2.311 (https://github.com/CJ-Chen/TBtools-II).

## Supporting information

S1 Fig Tagged versions of the H3.1, H3.2, and CENH3 and GBP genes were inserted into the *TK* locus in *Cryptosporidium parvum.* (A) A diagram of the TK-H3.1-3HA-Nluc-Neo-TK and TK-H3.2-3HA-Nluc-Neo-TK targeting vector and the expected sizes for the 5’ insertion site (490 and 530 bp, respectively) and 3’ insertion site (1091 bp) amplicons used to confirm proper insertion by PCR. (B) PCR analysis of H3.1-3HA and H3.2-3HA oocysts amplified in GKO mice. DNA purified from fecal samples collected from each mouse and a wild type (WT) control was used to amplify products specific for the 5′ and 3’ insertion sites of the integrated construct. (C) A diagram of the TK-3HA-CENH3-P2A-GBP-3Ty-Nluc-Neo-TK targeting vector and the expected sizes for the 5’ UTR (355 bp) and 3’ UTR (1091 bp) amplicons used to confirm proper insertion by PCR. (D) PCR analysis of TK-3HA-CENH3-P2A-GBP-3Ty-TK oocysts amplified in three NSG mice. DNA purified from fecal samples collected from each mouse (M1-3) and a wild type (WT) control was used to amplify products specific for the 5′ and 3’ insertion sites of the integrated construct.
(PDF)

S2 Fig The GBP and CENH3 genes were successfully tagged with HA tag and/or a spaghetti monster HA tag in C*ryptosporidium parvum.* (A) A diagram of the CENH3-3HA-Nluc-Neo, CENH3-smHA-Nluc-Neo, and GBP-3HA-Nluc-Neo targeting vectors and the expected sizes for the 5’ insertion site (492, 290, and 806 bp, respectively) and 3’ insertion site (591 and 326 bp, respectively) amplicons used to confirm proper insertion by PCR. (B) PCR analysis of CENH3-3HA, CENH3-smHA and GBP-3HA oocysts amplified in NSG mice. DNA purified from fecal samples collected from each mouse and a wild type (WT) control was used to amplify products specific for the 5′ and 3’ insertion sites of the integrated construct. (C) HCT-8 cells were infected with CENH3-smHA Cp parasites, fixed at 22 hpi, and stained with rat anti-HA followed by secondary antibodies Alexa Fluor 488 goat anti-rat IgG and Alexa Fluor 647 Streptavidin, followed by Hoechst staining. Scale bars, 1 μm.
(PDF)

S3 Fig Staining patterns of Centrin-1 in *Cryptosporidium parvum.* HCT-8 cells were infected with excysted *C. parvum* sporozoites for 2 h and then washed twice with PBS to remove extracellular parasites. Cultures were fixed at 9.5 hpi to capture trophozoites with one nucleus, early meronts with two nuclei, mid-stage meronts with four nuclei, and late-stage meronts with eight nuclei during the first round of merogony. Cell cultures were stained with a rabbit anti-centrin-1 primary antibody, followed by an Alexa Fluor 568-conjugated goat anti-rabbit IgG secondary antibody and Hoechst. Representative microscopy images showing the centrin-1 staining patterns in trophozoites with one nucleus (A), early meronts with two nuclei (B), mid-stage meronts with four nuclei (C), and late-stage meronts with eight nuclei (D). Scale bars, 1 μm. (E) Quantification of parasites exhibiting different centrin-1 staining patterns within a parasite vacuole. Each data point represents the number of parasites quantified from 10 images across three biological replicates, with two technical replicates per biological replicate. In total, > 370 parasites were used for the analysis.
(PDF)

S4 Fig Colocalization of GBP and CENH3 signals in *Cryptosporidium parvum.* (A-D) Representative microscopy images showing the overlap between the 488-nm channel, detecting CENH3-3HA with anti-HA primary antibody and Alexa Fluor 488-conjugated secondary antibody, and the 568-nm channel, detecting GBP-3Ty with anti-Ty primary antibody and Alexa Fluor 568-conjugated secondary antibody. Each asexual stage is shown with five different representative images, including trophozoites with one nucleus (A), early meronts with two nuclei (B), mid-stage meronts with four nuclei (C), and late-stage meronts with eight nuclei (D). The numbers in the upper right corner of the merged images indicate the conventional Pearson correlation coefficient calculated from pixel-intensity correlations, reflecting the degree of colocalization between CENH3 and GBP signals. Scale bars, 2 μm.
(PDF)

S5 Fig Additional images of centrin and tubulin staining in *Cryptosporidium parvum.* (B, C) HCT-8 cells were infected with wildtype parasites, fixed at 6.5 hpi to capture early meronts (2 nuclei), and stained with rabbit anti-centrin-1 and mouse anti-tubulin, followed by the secondary antibodies Alexa Fluor 488 goat anti-mouse IgG, Alexa Fluor 568 goat anti-rabbit IgG, and Hoechst. Meronts shown in panels B and C are identical to the meronts found in Fig 4C and D, respectively, but additional single-channel panels are shown here. Images were acquired as Z-stacks using LSCM-A and are presented with orthogonal views. Scale bars, 1 μm.
(PNG)

S6 Fig Genome-wide distribution of CENH3 in *Toxoplasma gondii.* (A) Genome browser view of *T. gondii* chromosome Ib showing CUT&RUN signals for CENH3-HA (HA antibody), H3K9me3, and H3K4me3. CENH3 signal enrichment positively correlates with H3K9me3 and inversely with H3K4me3. Peaks of CENH3 enrichment are indicated (dashed box). (B) Zoom in genome browser view of the dashed box in (A). (C) Genome browser view of all 13 *T. gondii* chromosomes showing CUT&RUN signals for CENH3-HA, with CENH3 peaks indicated (red arrows). (D) Schematic workflow for identifying centromeric windows. (E) List of centromeric windows identified in (D), corresponding to the peaks observed in the genome browser.
(PDF)

S7 Fig Genome-wide distribution of CENH3 in *Cryptosporidium parvum.* (A) Genome browser view of chromosome 1 showing CENH3-HA CUT&RUN profiles at different digestion times (5, 30, 60, and 120 min). (B) Schematic workflow for identifying centromeric windows (dashed box), including additional filtering based on WT-HA signal patterns. (C) Heatmaps of CUT&RUN signals within a 400 bp window around all 423 CENH3-enriched windows identified using the stepwise strategy showing in (B, dashed box). CUT&RUN signals for CENH3-HA *C. parvum* captured with HA antibody, wild type *C. parvum* captured with HA antibody (referred to as WT-HA), and wild type *C. parvum* captured with H3K9me3, and H3K4me3 antibodies. Each row represents an individual centromeric site, ranked by signal intensity. (D) Average signal profiles for CENH3-HA (green), WT-HA (gray), H3K9me3 (orange), and H3K4me3 (teal) across the 400 bp window around the 423 CENH3-enriched windows. (E) Chromosomal distribution of the 347 CENH3-enriched windows of *C. parvum* chromosomes (Chr#) with the additional filtering based on removing WT-HA signal. Numbers below each chromosome indicate the number of windows. (F, G) Evolutionary constraint analysis of CENH3-associated regions using nucleotide diversity (Pi) and dN/dS values. Comparisons were performed across *C. parvum* IIa and IId isolates (referred to as Cp). (F) Horizontal lines indicate the mean Pi values of genome-wide 400-bp sliding windows, used as a reference for comparison. Approximately 82% of CENH3-associated regions showed reduced Pi values when compared to the genome as a whole. (G) Horizontal lines indicate the mean dN/dS value of all orthologous genes with P < 0.05, used as a reference for comparison. Only one gene within the CENH3-associated regions showed elevated dN/dS relative to the genome-wide distribution. (H) Schematic workflow for identifying centromeric windows located in intergenic regions.
(PDF)

S1 Table Transgenic *Cryptosporidium* strains used in this study. (DOCX)

S2 Table Key Resources used in the experiments. (DOCX)

S3 Table Oligonucleotides and plasmids used in this study. (DOCX)

S1 Data Information of sequencing samples from the CUT&RUN and summary statistics of the data. (XLSX)

S2 Data Genomic location of the CENH3 sites within coding regions. (XLSX)

S3 Data Genomic location of the CENH3 sites within intergenic regions. (XLSX)

S1 File Raw images. (PDF)
