# Supplementary material for: Evidence for holocentric centromeres in the early branching apicomplexan parasite Cryptosporidium parvum
Source: PLoS Pathog. 2026 Oct 26;22(10):e1014708. doi: 10.1371/journal.ppat.1014708 (PMC13739221; doi:10.1371/journal.ppat.1014708)
Supplement: Supplementary file 1 [file ppat.1014708.s001.pdf]

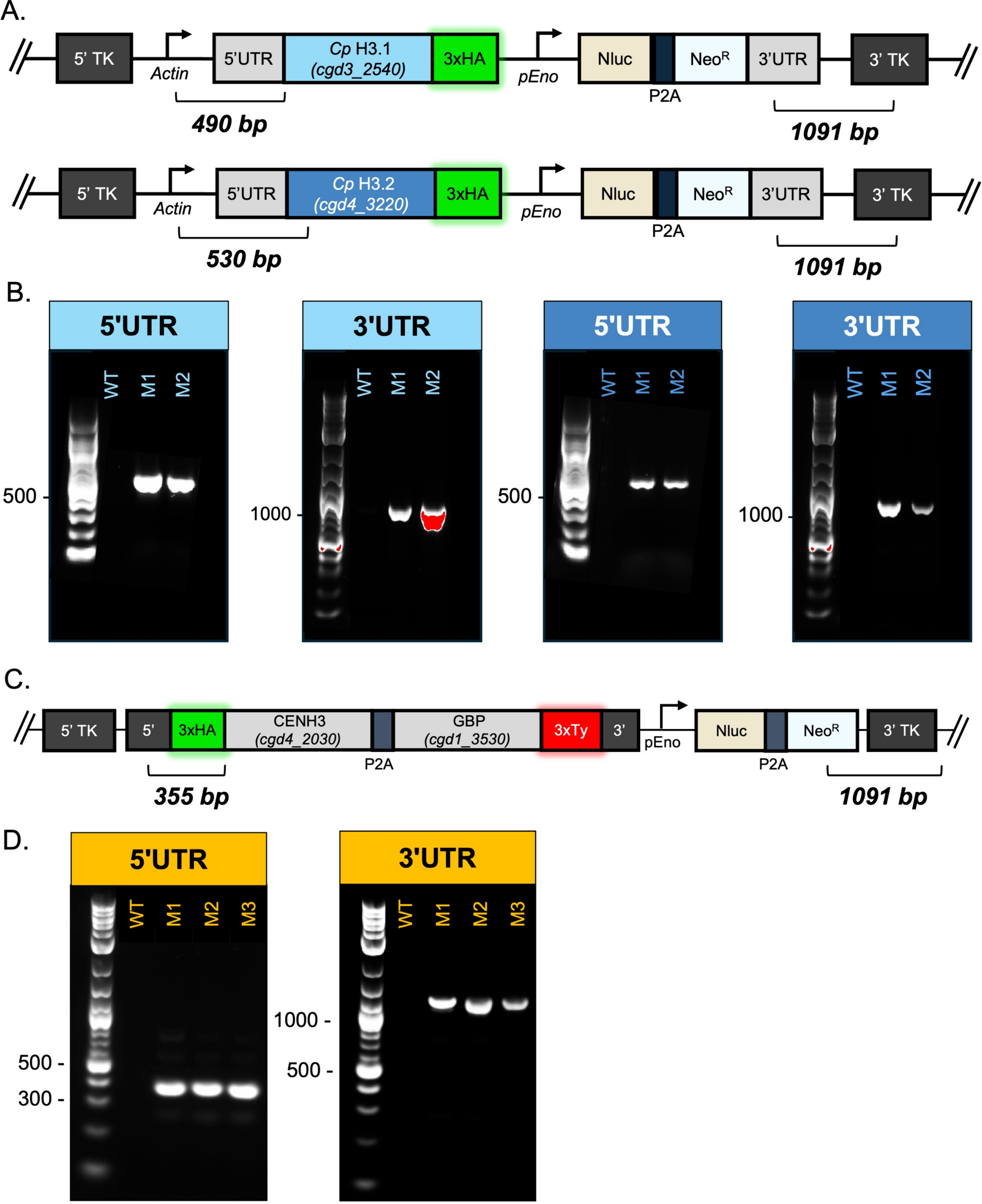

A.
5' TK
Actin
5'UTR
Cp H3.1 (cgd3_2540)
3xHA
pEno
Nluc
P2A
NeoR
3'UTR
3' TK
490 bp
1091 bp
Cp H3.2 (cgd4_3220)
530 bp
B.
5'UTR
3'UTR
WT M1 M2
500
1000
C.
5'
3xHA
CENH3 (cgd4_2030)
GBP (cgd1_3530)
3xTy
3'
355 bp
D.
WT M1 M2 M3
300
