# Supplementary material for: Evidence for holocentric centromeres in the early branching apicomplexan parasite Cryptosporidium parvum
Source: PLoS Pathog. 2026 Oct 26;22(10):e1014708. doi: 10.1371/journal.ppat.1014708 (PMC13739221; doi:10.1371/journal.ppat.1014708)
Supplement: Supplementary file 2 [file ppat.1014708.s002.pdf]

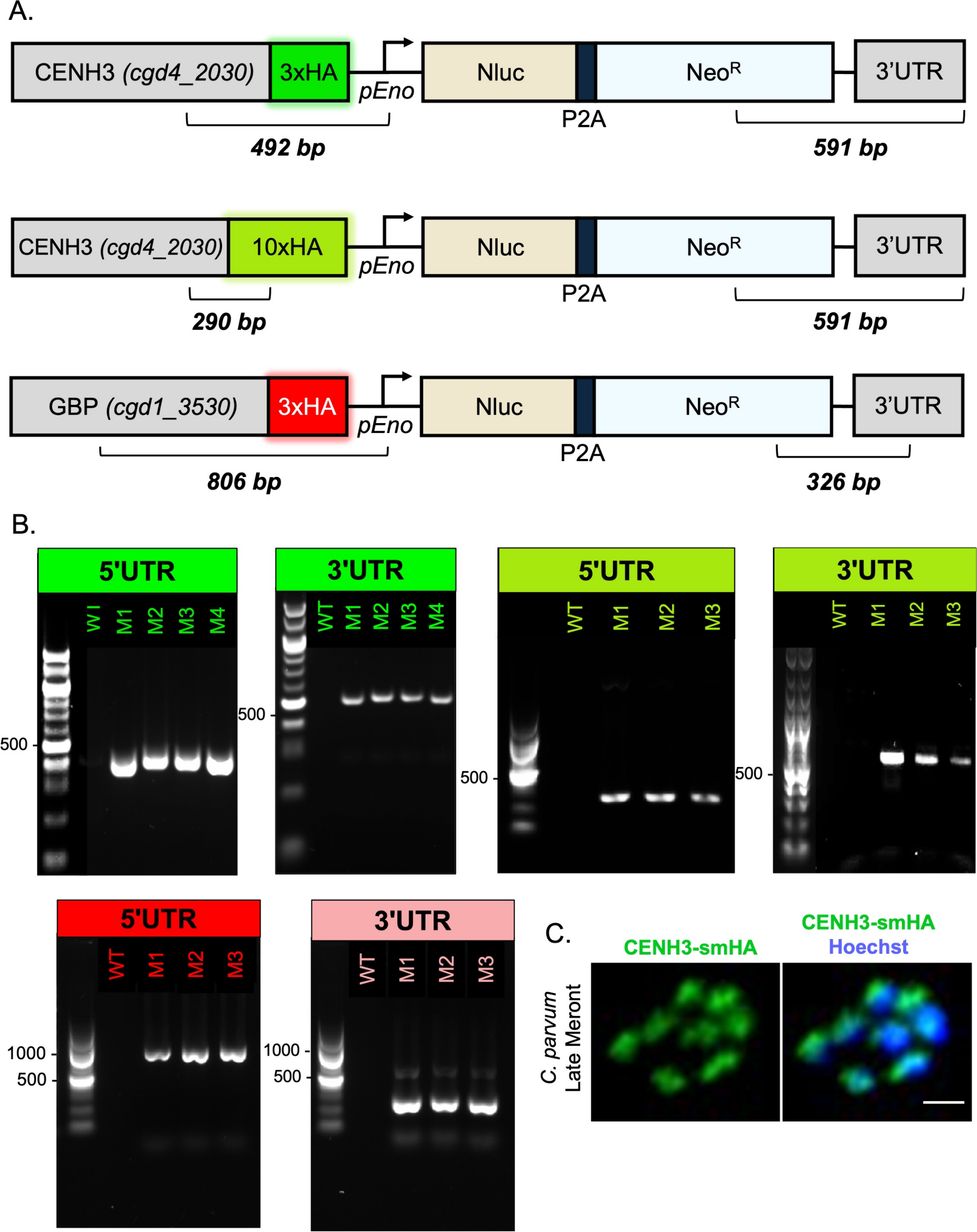
A.
CENH3 (cgd4_2030)
3xHA
pEno
Nluc
P2A
NeoR
3'UTR
492 bp
591 bp
CENH3 (cgd4_2030)
10xHA
pEno
Nluc
P2A
NeoR
3'UTR
290 bp
591 bp
GBP (cgd1_3530)
3xHA
pEno
Nluc
P2A
NeoR
3'UTR
806 bp
326 bp
B.
5'UTR
WT M1 M2 M3 M4
500
3'UTR
WT M1 M2 M3 M4
500
5'UTR
WT M1 M2 M3
500
3'UTR
WT M1 M2 M3
500
5'UTR
WT M1 M2 M3
1000
500
3'UTR
WT M1 M2 M3
1000
500
C.
CENH3-smHA
CENH3-smHA
Hoechst
C. parvum
Late Meront
