# Supplementary material for: Evidence for holocentric centromeres in the early branching apicomplexan parasite Cryptosporidium parvum
Source: PLoS Pathog. 2026 Oct 26;22(10):e1014708. doi: 10.1371/journal.ppat.1014708 (PMC13739221; doi:10.1371/journal.ppat.1014708)
Supplement: Supplementary file 3 [file ppat.1014708.s003.pdf]

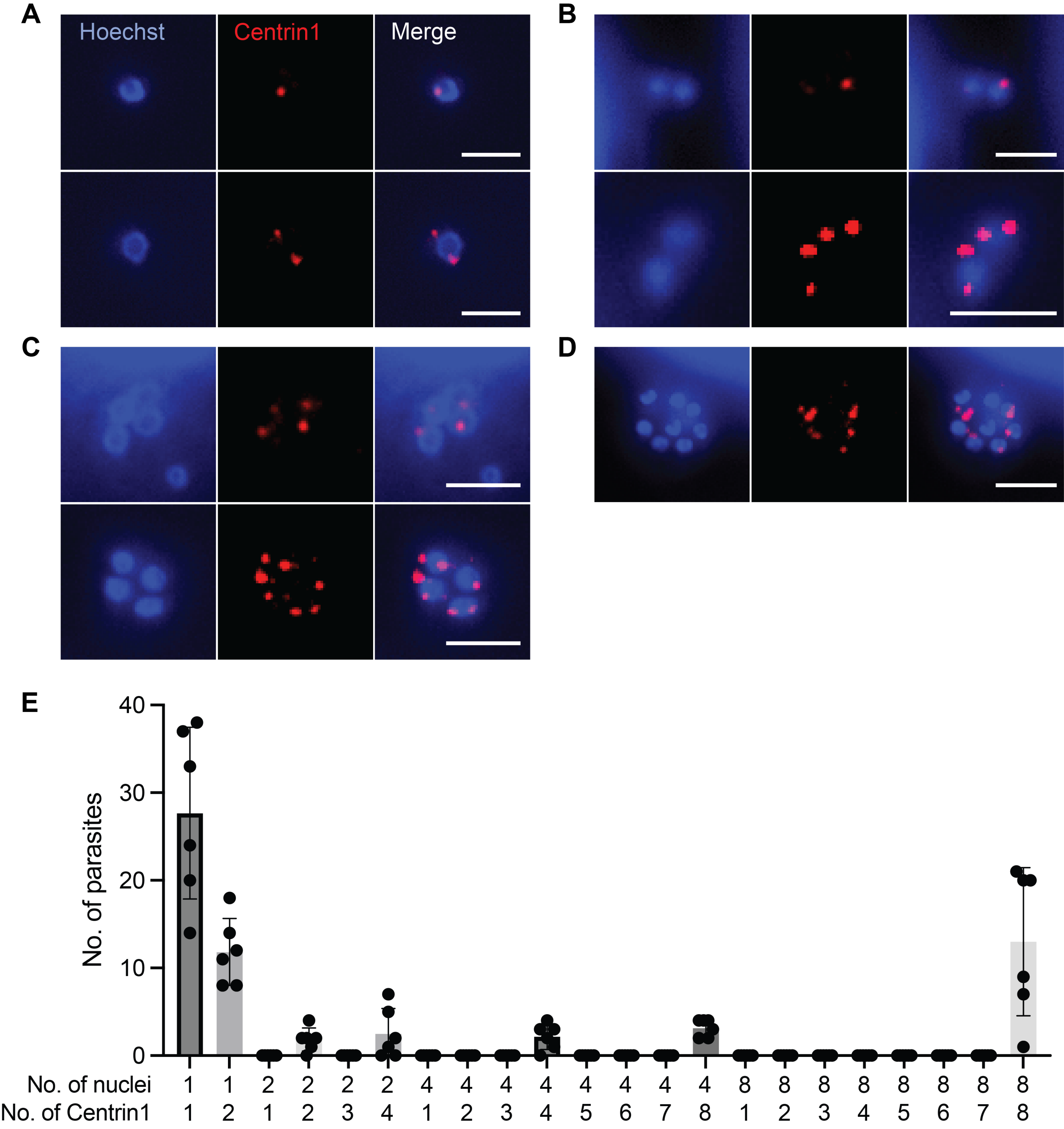

A
Hoechst
Centrin1
Merge
B
C
D
E
No. of parasites
40
30
20
10
0
No. of nuclei 1 1 2 2 2 2 4 4 4 4 4 4 4 4 8 8 8 8 8 8 8 8
No. of Centrin1 1 2 1 2 3 4 1 2 3 4 5 6 7 8 1 2 3 4 5 6 7 8
