# Supplementary material for: Evidence for holocentric centromeres in the early branching apicomplexan parasite Cryptosporidium parvum
Source: PLoS Pathog. 2026 Oct 26;22(10):e1014708. doi: 10.1371/journal.ppat.1014708 (PMC13739221; doi:10.1371/journal.ppat.1014708)
Supplement: Supplementary file 4 [file ppat.1014708.s004.pdf]

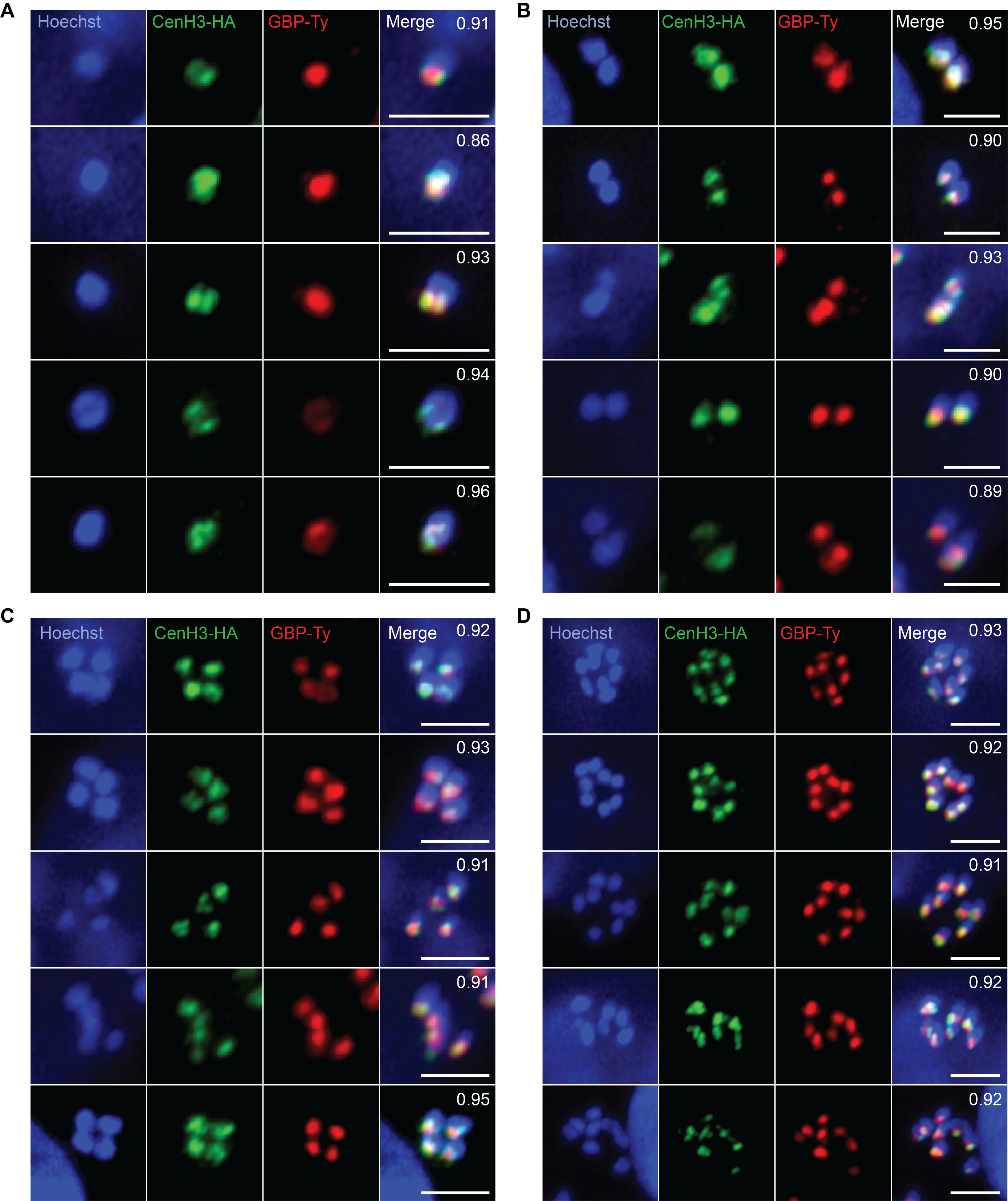
A
Hoechst
CenH3-HA
GBP-Ty
Merge
0.91
0.86
0.93
0.94
0.96
B
Hoechst
CenH3-HA
GBP-Ty
Merge
0.95
0.90
0.93
0.90
0.89
C
Hoechst
CenH3-HA
GBP-Ty
Merge
0.92
0.93
0.91
0.91
0.95
D
Hoechst
CenH3-HA
GBP-Ty
Merge
0.93
0.92
0.91
0.92
0.92
