# Supplementary material for: Evidence for holocentric centromeres in the early branching apicomplexan parasite Cryptosporidium parvum
Source: PLoS Pathog. 2026 Oct 26;22(10):e1014708. doi: 10.1371/journal.ppat.1014708 (PMC13739221; doi:10.1371/journal.ppat.1014708)
Supplement: Supplementary file 5 [file ppat.1014708.s006.pdf]

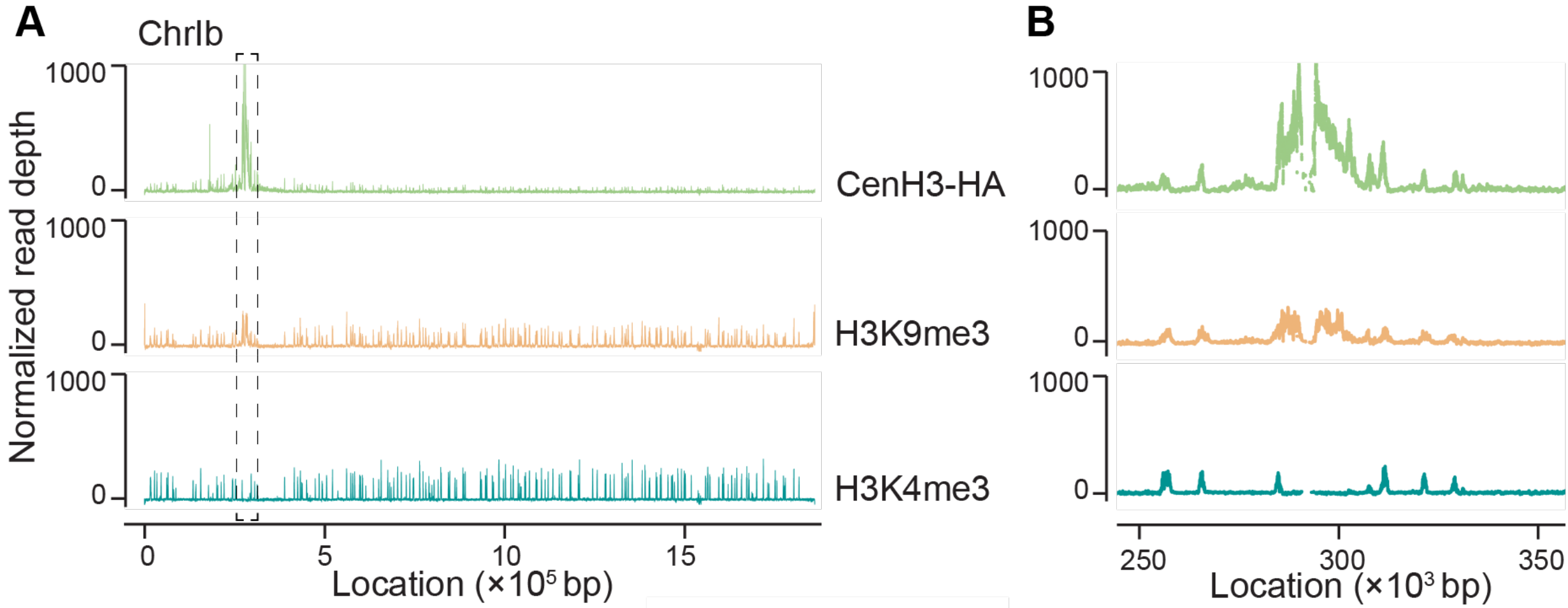

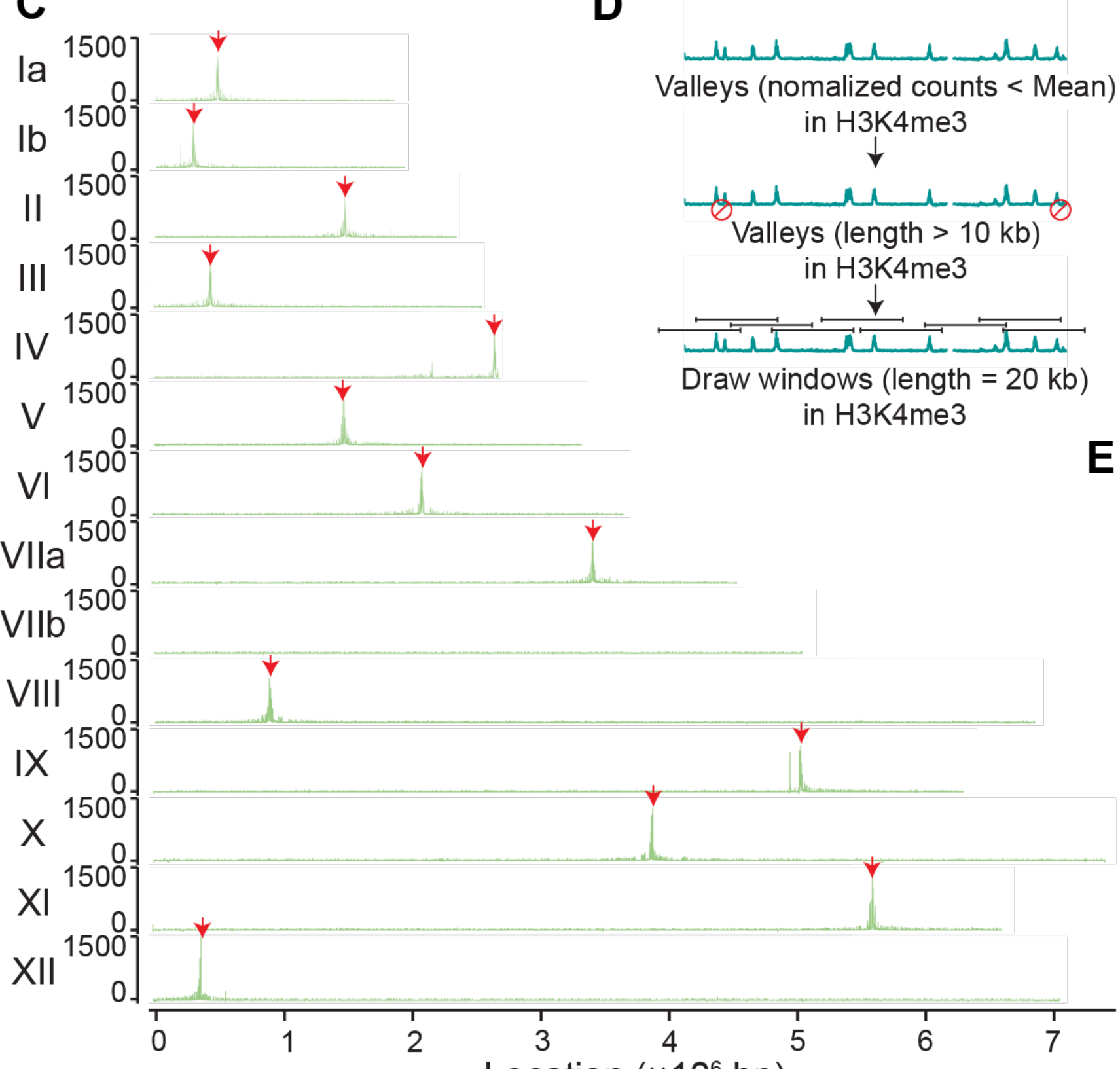

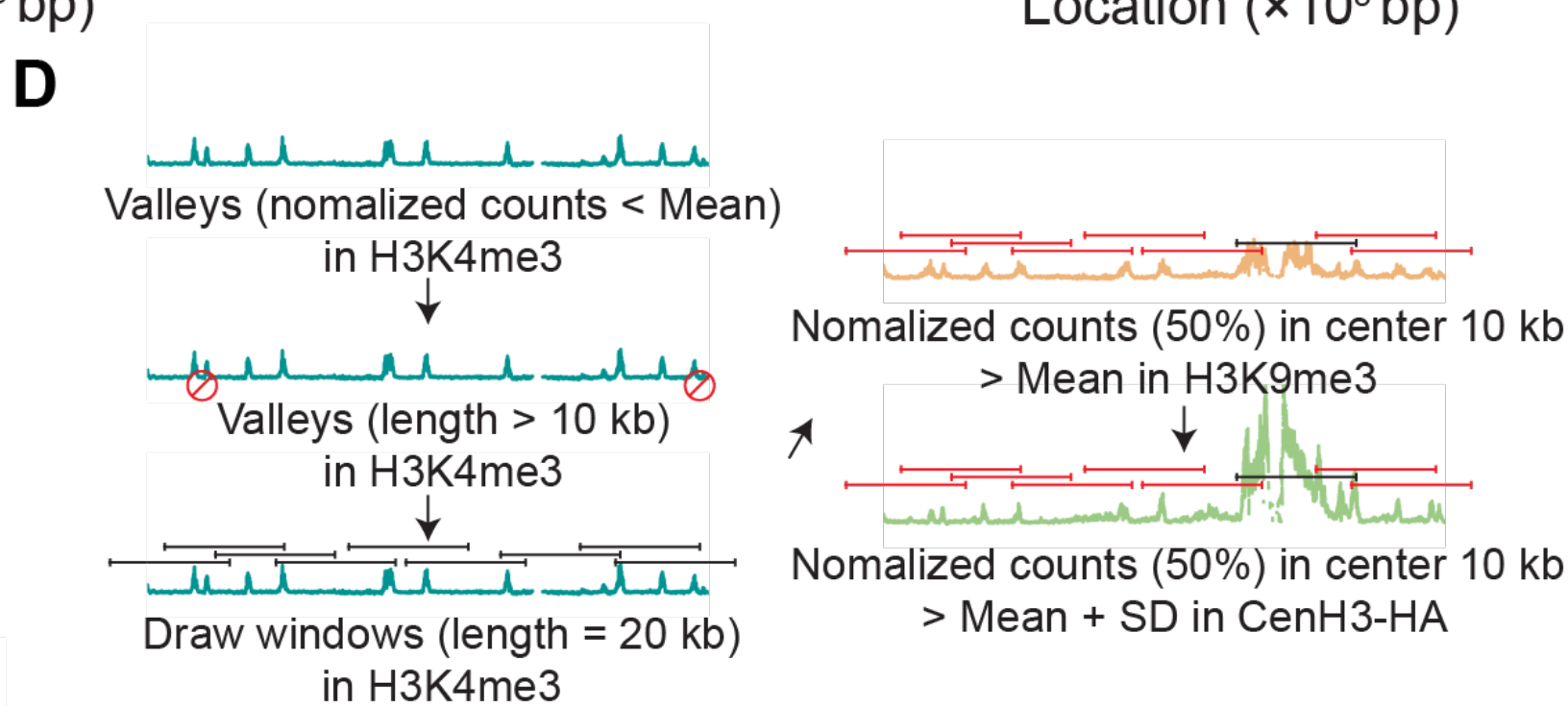

E

	Start	End
chrIa	474463	494463
chrIb	284200	304200
chrII	1473350	1493350
chrIII	425583	445583
chrIV	2637094	2657094
chrV	1470349	1490349
chrVI	2083408	2103408
chrVIIa	3407284	3427284
chrVIIb		
chrVIII	894710	914710
chrIX	5049540	5069540
chrX	3909688	3929688
chrXI	5582488	5602488
chrXII	356884	376884
