# Supplementary material for: Evidence for holocentric centromeres in the early branching apicomplexan parasite Cryptosporidium parvum
Source: PLoS Pathog. 2026 Oct 26;22(10):e1014708. doi: 10.1371/journal.ppat.1014708 (PMC13739221; doi:10.1371/journal.ppat.1014708)
Supplement: Supplementary file 6 [file ppat.1014708.s007.pdf]

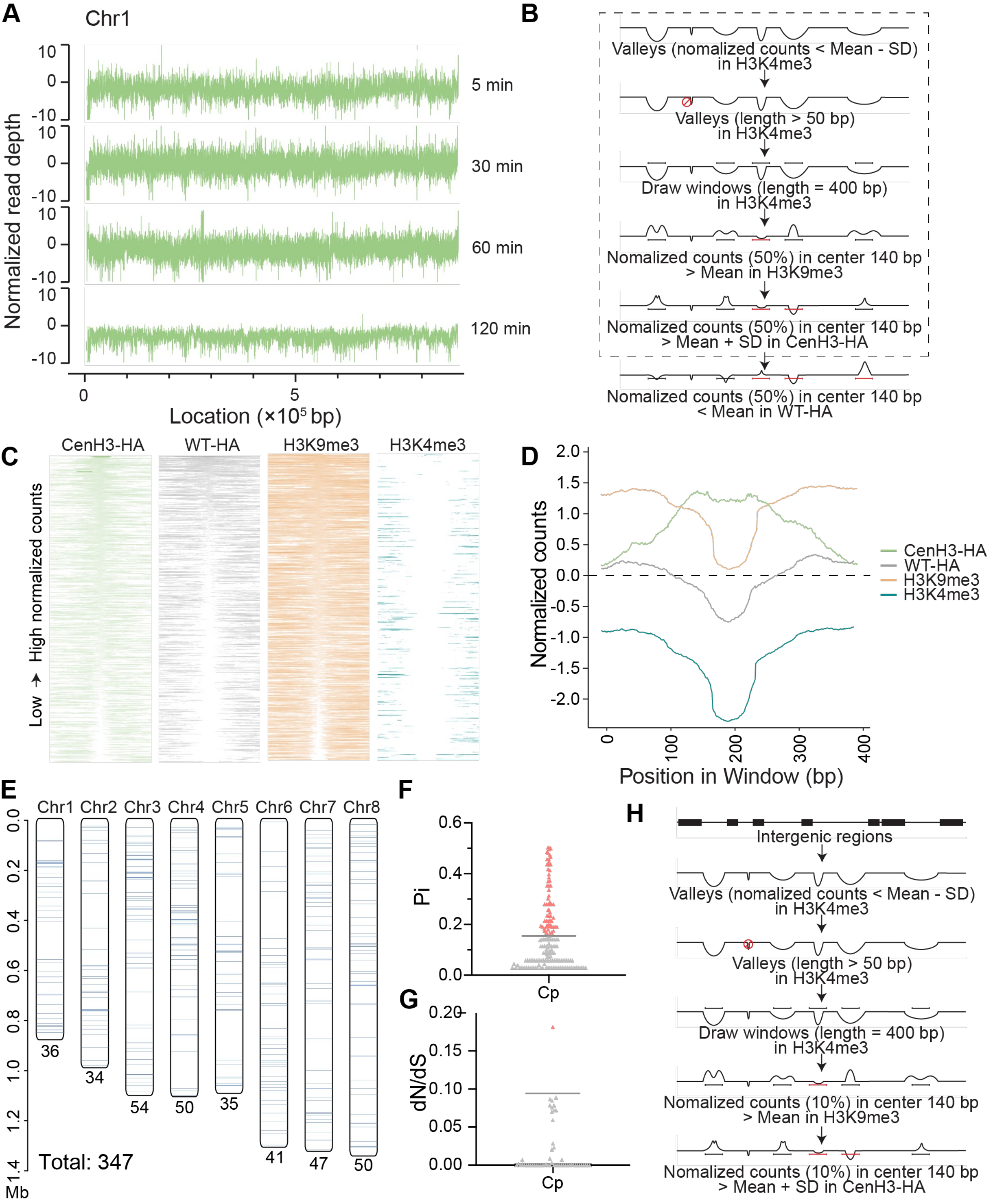

A
Chr1
Normalized read depth
5 min
30 min
60 min
120 min
Location (×10^5 bp)
B
Valleys (nomalized counts < Mean - SD) in H3K4me3
Valleys (length > 50 bp) in H3K4me3
Draw windows (length = 400 bp) in H3K4me3
Nomalized counts (50%) in center 140 bp > Mean in H3K9me3
Nomalized counts (50%) in center 140 bp > Mean + SD in CenH3-HA
Nomalized counts (50%) in center 140 bp < Mean in WT-HA
C
CenH3-HA
WT-HA
H3K9me3
H3K4me3
Low → High normalized counts
D
Normalized counts
Position in Window (bp)
CenH3-HA
WT-HA
H3K9me3
H3K4me3
E
Chr1
Chr2
Chr3
Chr4
Chr5
Chr6
Chr7
Chr8
36
34
54
50
35
41
47
50
Total: 347
Mb
F
Pi
Cp
G
dN/dS
Cp
H
Intergenic regions
Valleys (nomalized counts < Mean - SD) in H3K4me3
Valleys (length > 50 bp) in H3K4me3
Draw windows (length = 400 bp) in H3K4me3
Nomalized counts (10%) in center 140 bp > Mean in H3K9me3
Nomalized counts (10%) in center 140 bp > Mean + SD in CenH3-HA
