# Supplementary material for: Evidence for holocentric centromeres in the early branching apicomplexan parasite Cryptosporidium parvum
Source: PLoS Pathog. 2026 Oct 26;22(10):e1014708. doi: 10.1371/journal.ppat.1014708 (PMC13739221; doi:10.1371/journal.ppat.1014708)
Supplement: Supplementary file 7 [file ppat.1014708.s014.pdf]

Final Figure - cropped and increased contrast

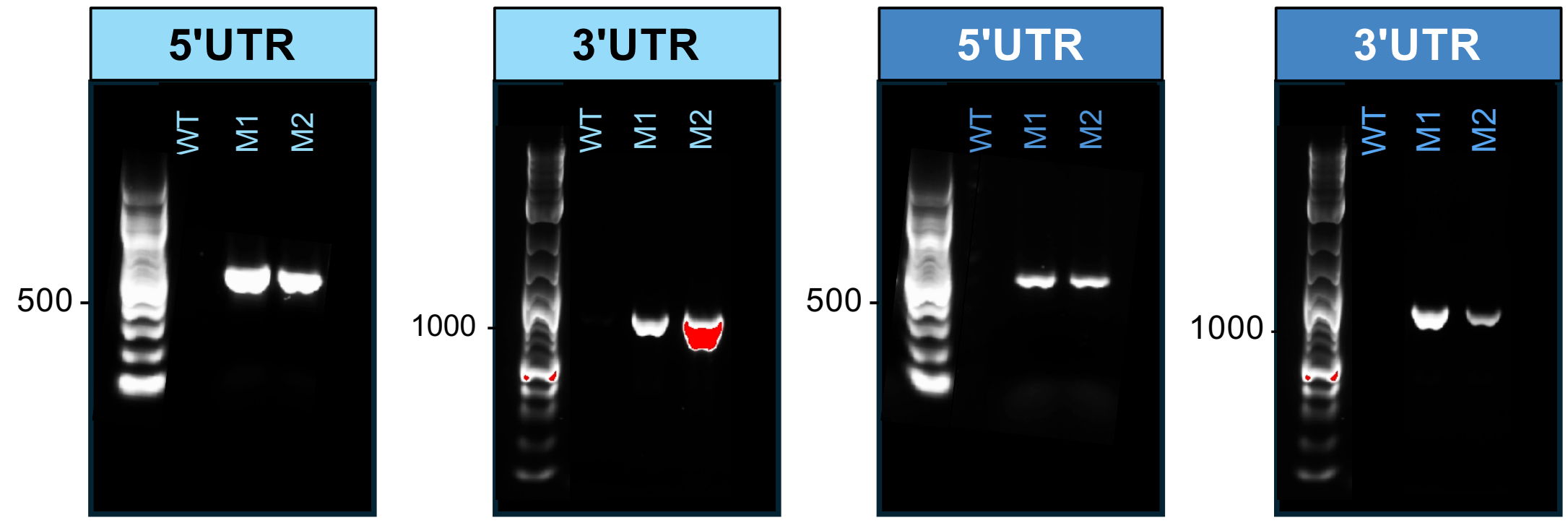

20241210 5' and 3' TK UTR ME97-ME99.jpg

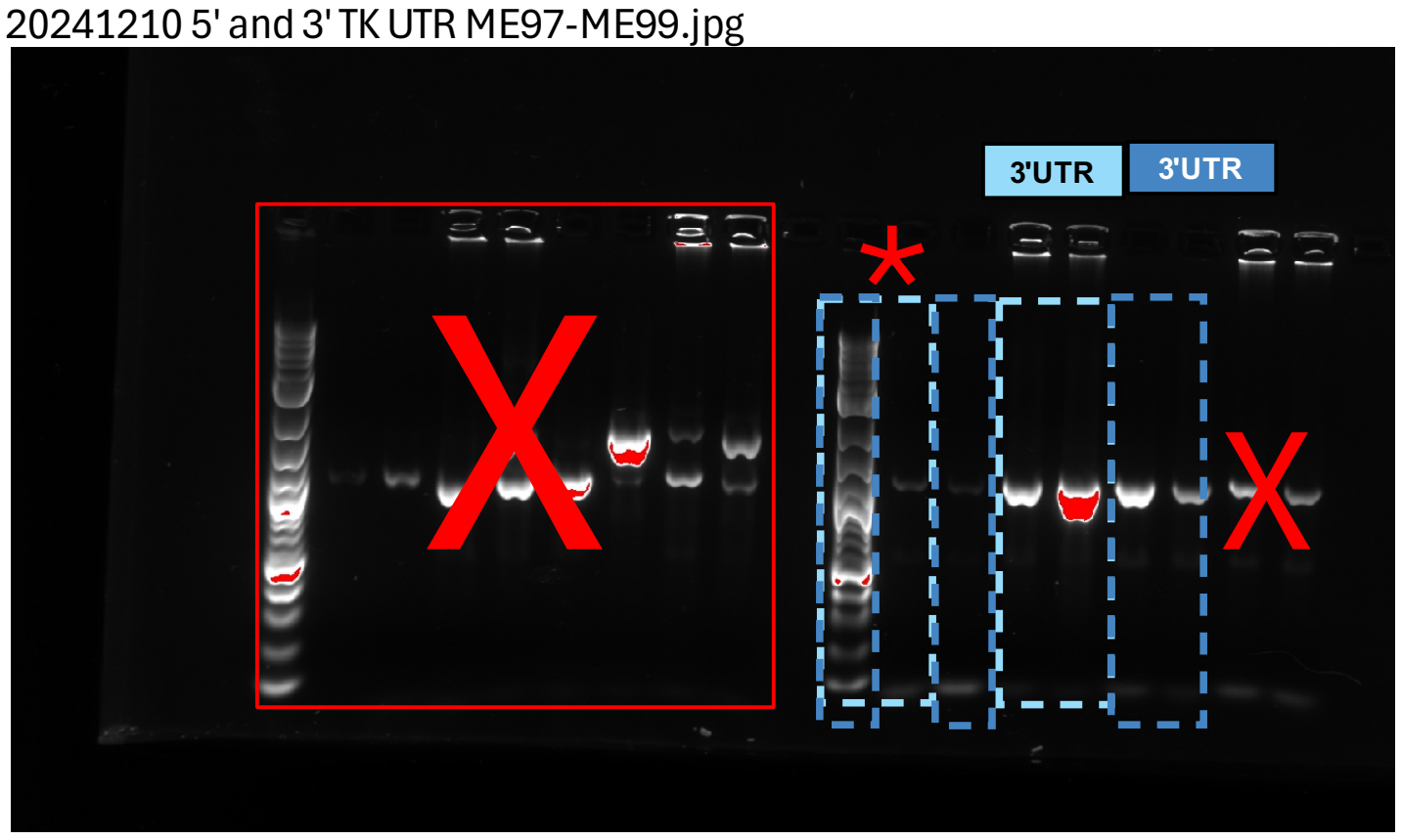

202501 TK H3.1, H3.2 5' with custom primers successful PCR.jpg

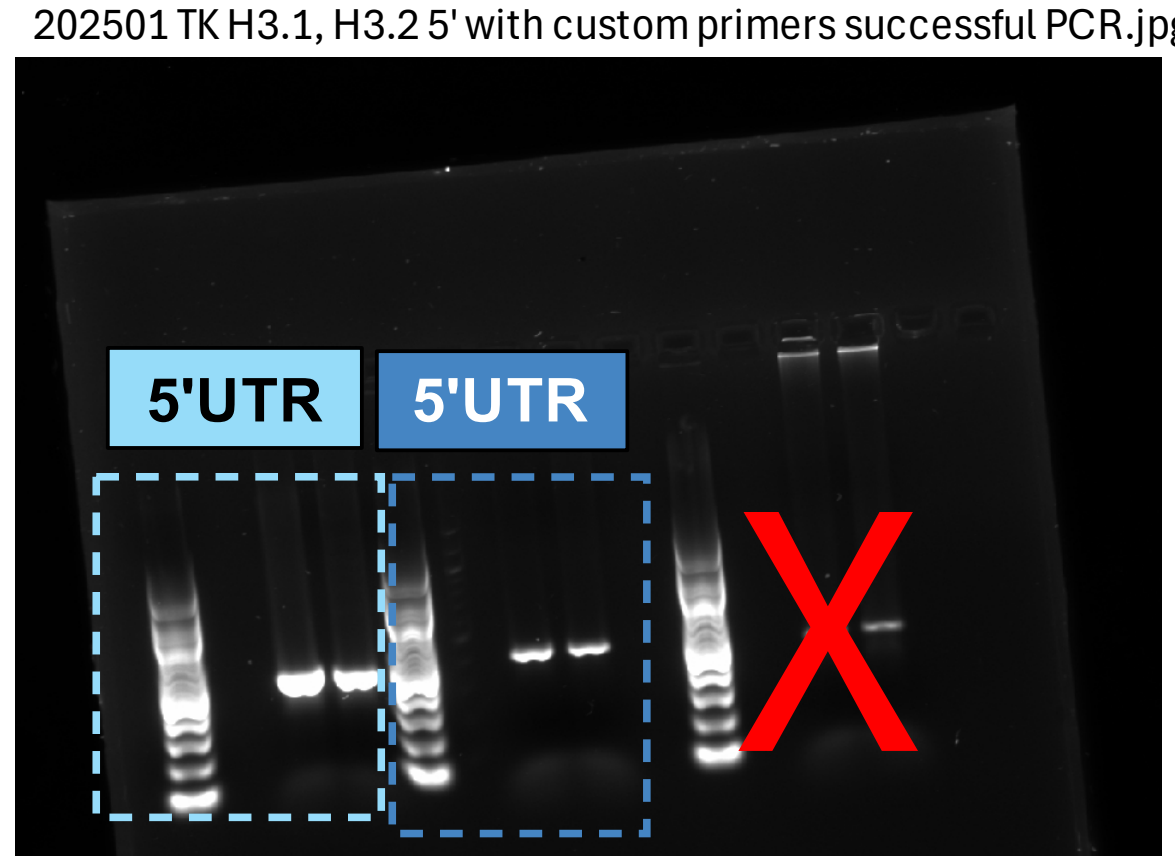

Key: WT = wildtype, M1/M2 = mouse 1,2,etc., 3'UTR/5'UTR= checking for proper insertion of the 3' end of insert and 5' end of insert

*For 3'UTR gel = Lane 1 is ladder, lane 2 is WT for 3'UTR H3.1, lane 3 is WT for 3'UTR H3.1, lanes 4 & 5 are 3'UTR for mouse samples for H3.1, and lanes 6 & 7 are 3'UTR for mouse samples for H3.2. Lane 8&9 are for a different experiment.

Final Figure - cropped and increased contrast

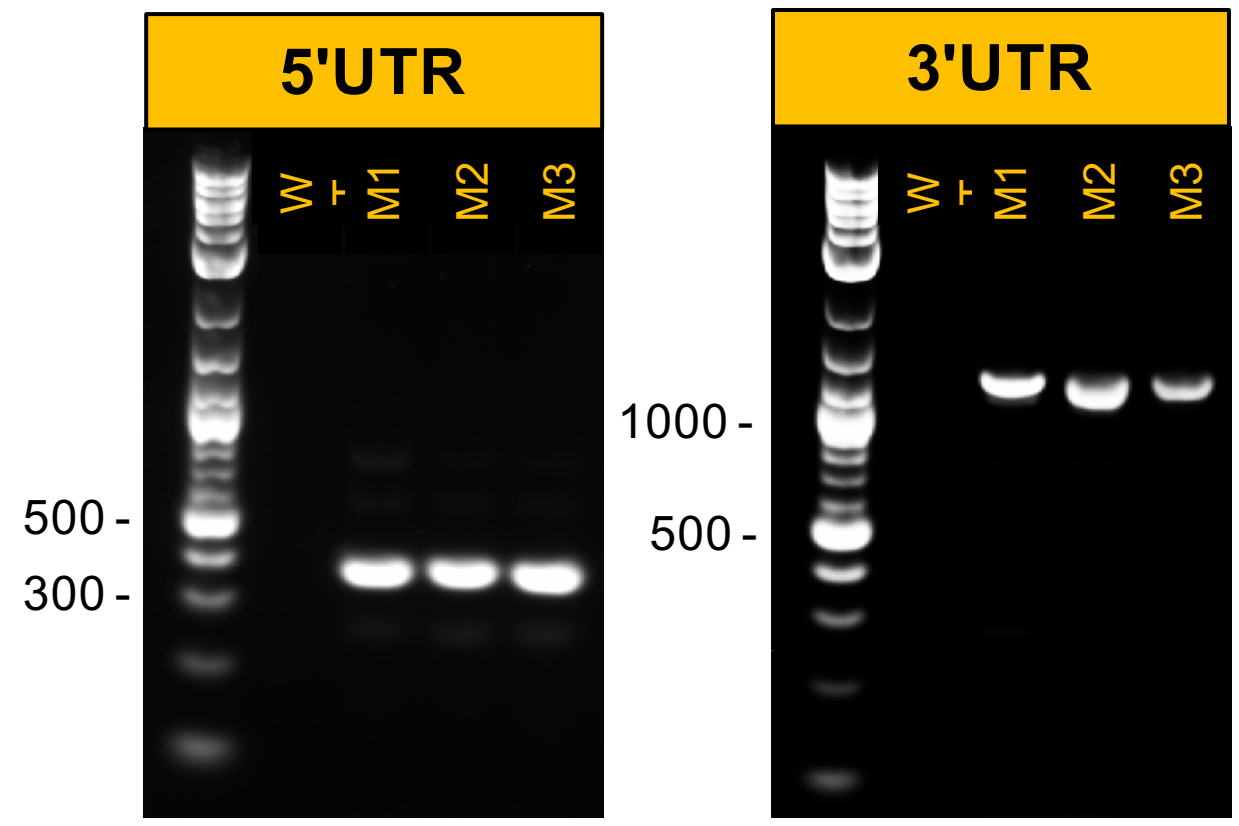

20230817 5' and 3' insert PCR D27 samples ME50.tif

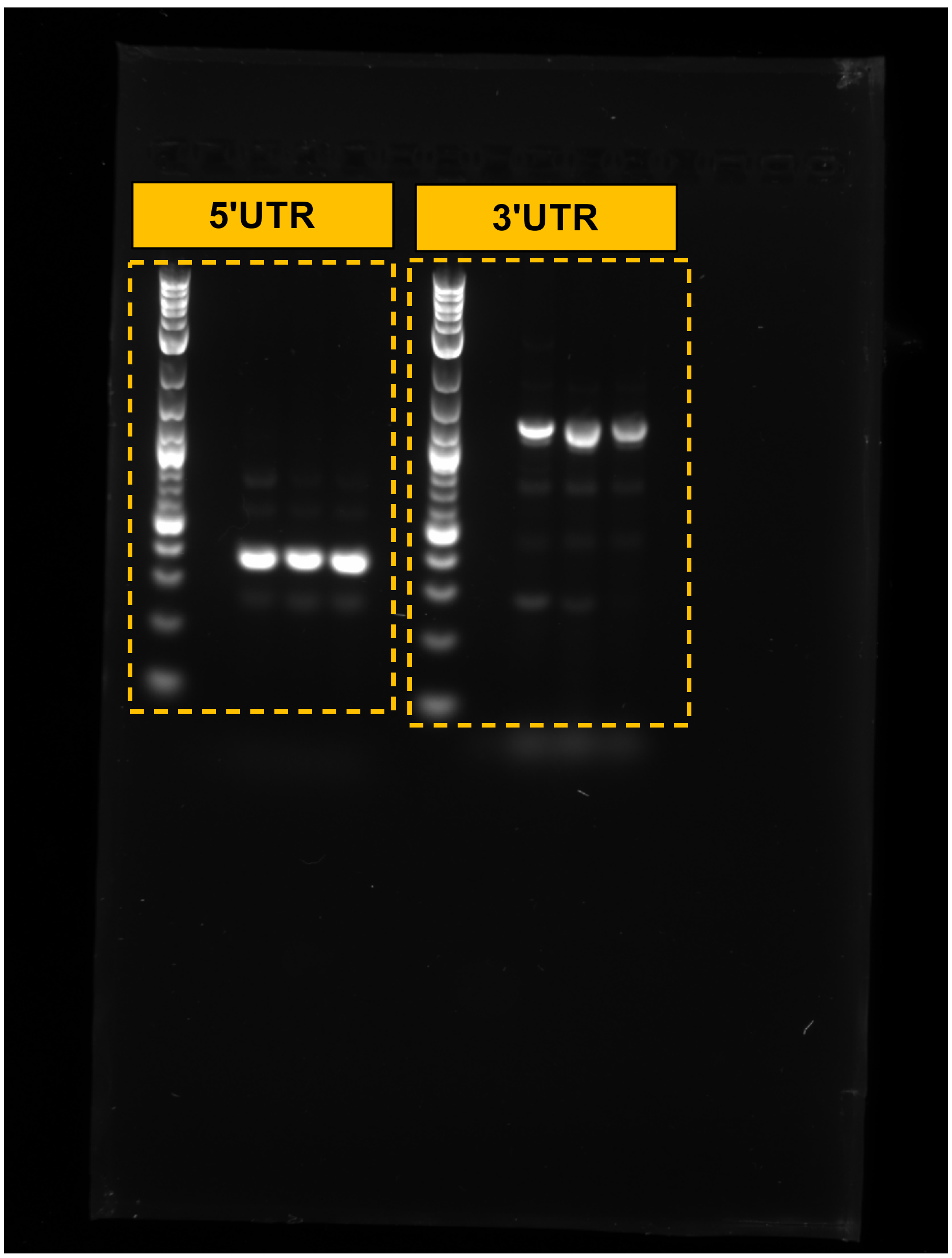

Key: WT = wildtype, M1/M2 = mouse 1,2,etc., 3'UTR/5'UTR= checking for proper insertion of the 3' end of insert and 5' end of insert

Final Figure - cropped and increased contrast

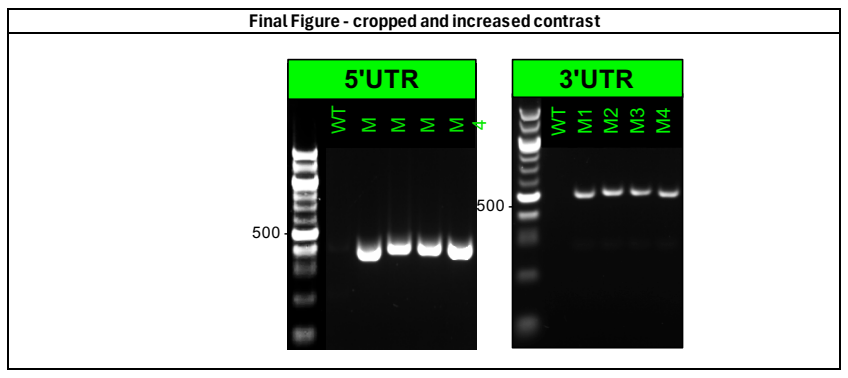

Final Figure - cropped and increased contrast

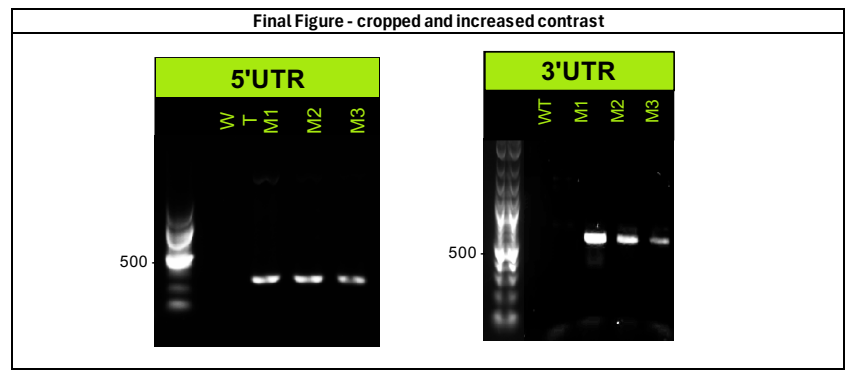

Final Figure - cropped and increased contrast

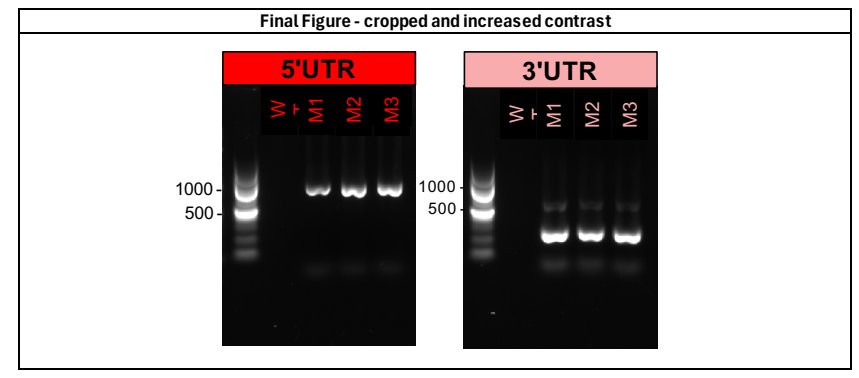

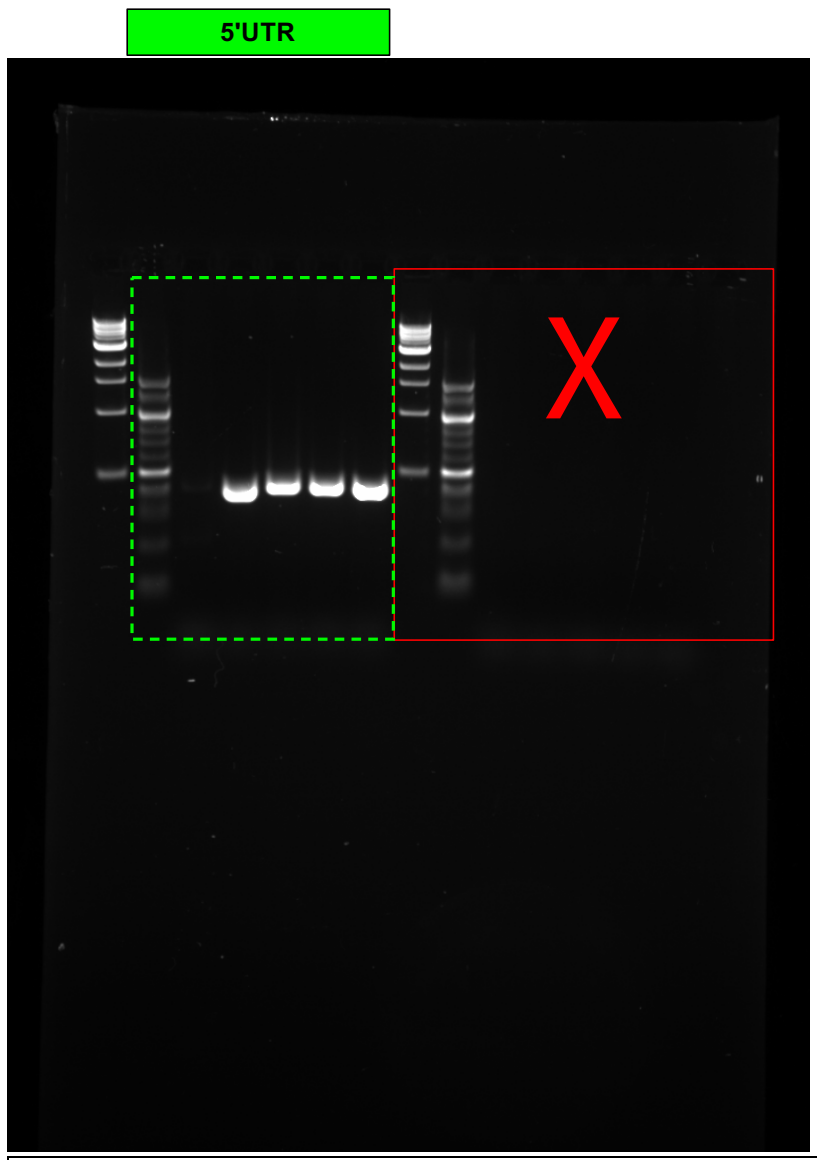

20220516 5' and 3' insert check gel.tif

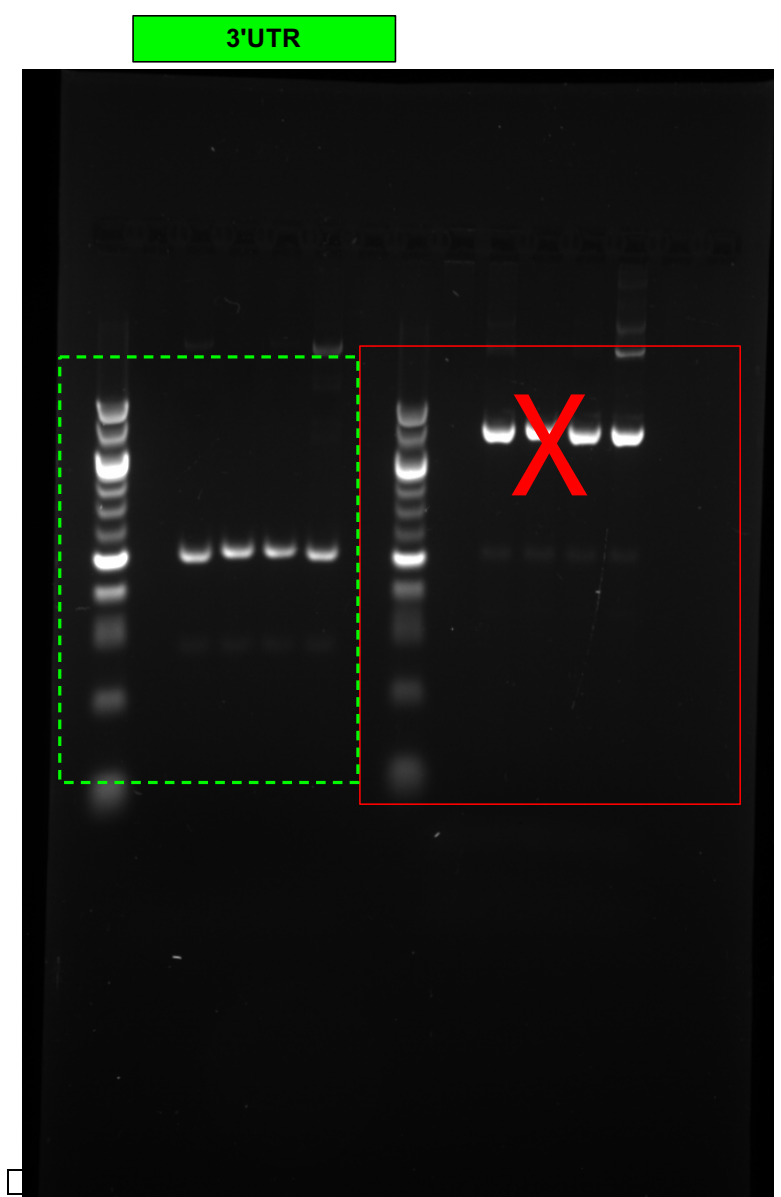

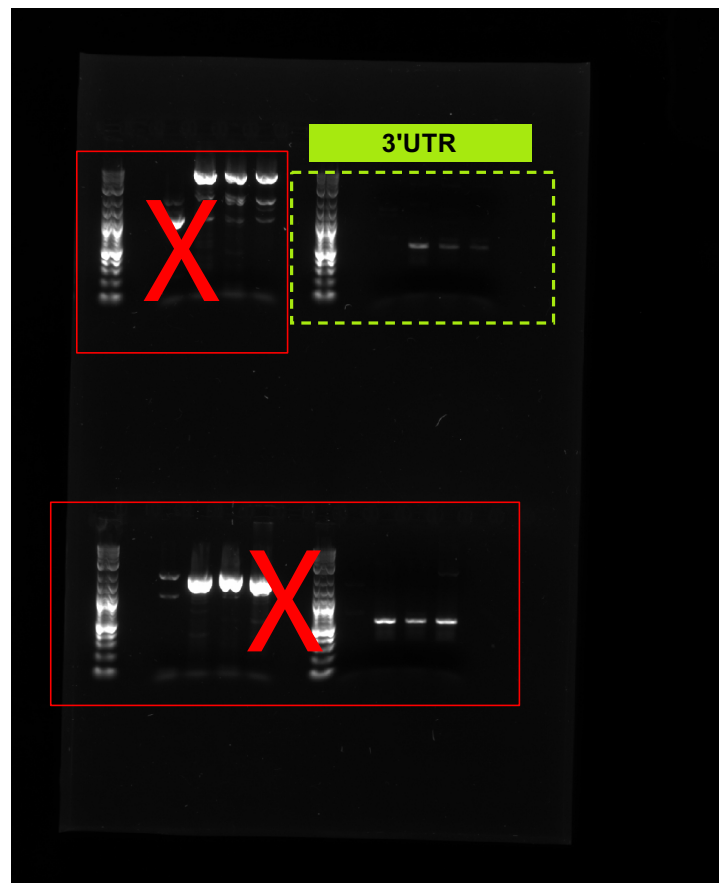

SUPPLEMENTAL 20240826 smHA 5 and 3 on top, GFP 5 and 3 on bottom.jpg

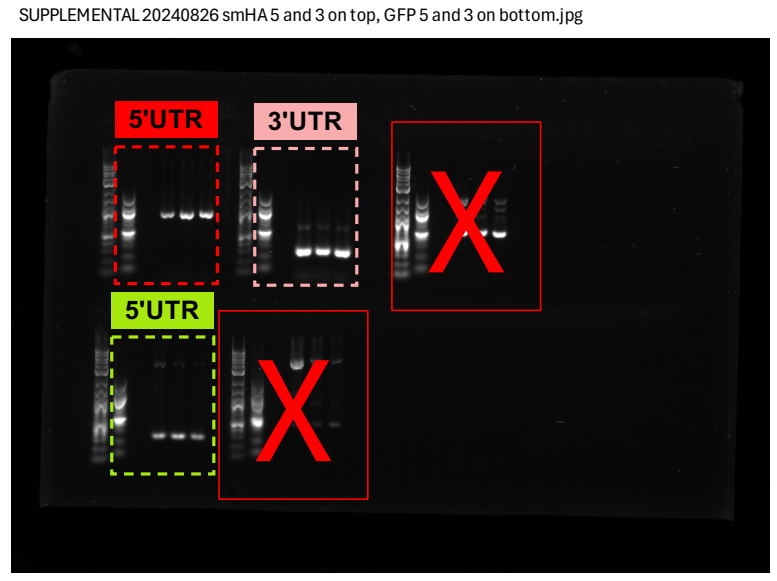

20240828 Gbp 5 and 3 UTR, CenH3 smHA pEnoR-pLinker F, and CenH3 F and F2 and pLinker R SUPPLEMENTAL 45 min

Key: WT = wildtype, M1/M2 = mouse 1,2,etc., 3'UTR/5'UTR= checking for proper insertion of the 3' end of insert and 5' end of insert
