# Supplementary material for: Evidence for holocentric centromeres in the early branching apicomplexan parasite Cryptosporidium parvum
Source: PLoS Pathog. 2026 Oct 26;22(10):e1014708. doi: 10.1371/journal.ppat.1014708 (PMC13739221; doi:10.1371/journal.ppat.1014708)
Supplement: Supplementary file 8 [file ppat.1014708.s008.docx]

**S1 Table.** Transgenic *Cryptosporidium* strains used in this study.

| **Line** | **Genotype** | **Use** | **Endogenous or second copy** | **Source** |
| --- | --- | --- | --- | --- |
| H3.1-3HA | TK-H3.1-3HA-Nluc-P2A-Neo^R^-TK | Epitope tagging for localization | Second copy | This study |
| H3.2-3HA | TK-H3.2-3HA-Nluc-P2A-Neo^R^-TK | Epitope tagging for localization | Second copy | This study |
| CENH3-3HA | CENH3-3HA-Nluc-P2A-Neo^R^ | Epitope tagging for localization, ExM, EM | Endogenous | This study |
| GBP-3HA | GBP-3HA-Nluc-P2A-Neo^R^ | Epitope tagging for localization | Endogenous | This study |
| 3HA-CENH3-GBP-Ty | TK-3HA-CENH3-P2A-GBP-3Ty-Nluc-P2A-Neo^R^-TK | Epitope tagging for localization | Second copy | This study |
| CENH3-smHA | CENH3-smHA-Nluc-P2A-Neo^R^ | CUT&RUN, ExM, EM | Endogenous | This study |
