# Supplementary material for: Evidence for holocentric centromeres in the early branching apicomplexan parasite Cryptosporidium parvum
Source: PLoS Pathog. 2026 Oct 26;22(10):e1014708. doi: 10.1371/journal.ppat.1014708 (PMC13739221; doi:10.1371/journal.ppat.1014708)
Supplement: Supplementary file 9 [file ppat.1014708.s009.docx]

**S2 Table.** Key Resources used in the experiments.

| **REAGENT or RESOURCE** | **SOURCE** | **IDENTIFIER** |
| --- | --- | --- |
| **Antibodies** | | |
| Rat monoclonal anti-HA (3F10) (IFA, ExM, and EM) | Sigma-Aldrich | Cat# 11867423001 |
| Rabbit polyclonal anti-HA (SG77) (ExM and EM only) | Novus Biologicals | Cat# 71-5500 |
| Polyclonal Rabbit anti-GFP (ExM and EM only) | Origene | TP401 |
| Polyclonal Rabbit anti-GFP (ExM and EM only) | ThermoFisher | Cat# A-11122 |
| Rabbit anti-phosphorylated Histone H3 (PH3) | Sigma-Aldrich | Cat# H0412 |
| Rabbit anti-Centrin | Kerafast | Cat# EBC004 |
| Mouse anti-tubulin (12G10) | Developmental Studies Hybridoma Bank | Antibody ID# AB_1157911 |
| Rabbit *Plasmodium falciparum* nuclear pore (NUP116) | The Laboratory of Dr. Artur Scherf [1] | *Pf*Nup116 |
| Mouse anti-Ty | In house hybridoma | mAb Clone BB2 |
| *Vicia Villosa* Lectin (VVL), Biotinylated | Vector Labs | Cat# B-1235-2 |
| Rat PanCp (ExM only) | In house antisera [2] |  |
| Alexa Fluor 488 goat anti-rat IgG (H+L) | Thermo Fisher Scientific | Cat# A11006 |
| Alexa Fluor 488 goat anti-rabbit IgG (H+L) (IFA and ExM) | Thermo Fisher Scientific | Cat# A11034 |
| Alexa Fluor 568 goat anti-rat IgG (H+L) (IFA and ExM) | Thermo Fisher Scientific | Cat# A11077 |
| Alexa Fluor 568 goat anti-rabbit IgG (H+L) | Thermo Fisher Scientific | Cat# A11011 |
| Alexa Fluor 647 goat anti-mouse IgG (H+L) | Thermo Fisher Scientific | Cat# A21235 |
| Alexa Fluor 647 Streptavidin | Thermo Fisher Scientific | Cat# S21374 |
| 18 nm colloidal gold–conjugated goat anti‑rabbit IgG (H+L) (EM only) | Jackson ImmunoResearch Laboratories Inc. | Cat# 111‑215‑144 |
| Hoechst | Thermo Fischer Scientific | Cat# C10637 |
| H3K9me2 | Sigma-Aldrich | Cat# SAB4200609 |
| H3K9me3 | Abcam | Cat# ab8898 |
| HA Tag CUTANA™ CUT&RUN Antibody | Epicypher | Cat# 13-2010 |
| ***Cryptosporidium* & Bacterial Strains** | | |
| *Cryptosporidium parvum* AUCP-1 isolate | Laboratory of William Witola | N/A |
| NEB 5-alpha Competent E. coli (High Efficiency) | New England Biolabs | Cat# C2987H |
| **Chemicals, Reagents & Supplies** | | |
| Sodium taurocholate hydrate | Sigma-Aldrich | Cat# 86339 |
| Sodium chloride | Sigma-Aldrich | Cat# S7653-250G |
| Tween-20 | Sigma-Aldrich | Cat# 11332465001 |
| Fetal bovine serum | Gibco | Cat# 10-082-147 |
| Paromomycin sulfate salt | Sigma-Aldrich | Cat# P9297 |
| Bovine serum albumin | Sigma-Aldrich | Cat# A7030 |
| Poly-L-Lysine solution (0.01%) | Sigma-Aldrich | Cat# P4707 |
| Triton X-100 | Thermo Fisher Scientific | Cat# BP151 |
| ProLong Glass Antifade Mountant | Thermo Fisher Scientific | Cat# P36984 |
| Formaldehyde (methanol-free), Ultrapure EM Grade | Polysciences, Inc. | Cat# 04018-1 |
| GelRed nucleic acid gel stain | Biotium | Cat# 41003-1 |
| Sodium bicarbonate | ATCC | Cat# 30-2002 |
| Carbenicillin disodium salt | Sigma-Aldrich | Cat# C3416 |
| Fetal bovine serum (FBS) | Sigma-Aldrich | Cat# F6178 |
| RPMI 1640 ATCC Modification medium | Thermo Fisher Scientific | Cat# A14091-01 |
| Dulbecco’s Phosphate-Buffered Saline (DPBS) | Invitrogen | Cat# 15575020 |
| Falcon 15 mL/50 mL Polystyrene Centrifuge Tubes | Corning Costar | Cat# 352099, 430290 |
| HEPES-KOH | Sigma-Aldrich | Cat# H0527 |
| MgCl2 | Sigma-Aldrich | Cat# 208337 |
| KCl | Sigma-Aldrich | Cat# P3911 |
| IGEPAL CA-630 | Sigma-Aldrich | Cat# I8896 |
| cOmplete EDTA-free Protease Inhibitor Cocktail | Sigma-Aldrich | Cat# 11873580001 |
| **Commercial Assays** | | |
| e-Myco plus Mycoplasma PCR detection kit | Boca Scientific | Cat#: 25237 |
| QIAamp DNA Mini kit | QIAGEN | Cat# 51306 |
| QIAamp Powerfecal Pro DNA kit | QIAGEN | Cat# 51804 |
| TB Green Advantage qPCR premix | Takara Bio | Cat# 639676 |
| Gibson Assembly Cloning kit | New England Biosciences | Cat# E5510S |
| Q5 Site-directed Mutagenesis kit | New England Biosciences | Cat# E0554S |
| Q5 Hot Start High-Fidelity 2X master mix | New England Biosciences | Cat# M0494S |
| SF Cell Line 4D-Nucleofector X Kit L | Lonza | Cat# V4XC-2024 |
| Nano-Glo Luciferase Assay kit | Promega | Cat# N1120 |
| CUTANA™ ChIC/CUT&RUN Kit | Epicypher | Cat# 14-1048 |
| CUTANA™ CUT&RUN Library Prep Kit | Epicypher | Cat# 14-1001 |
| **Experimental Models: Cell Lines** | | |
| Human: HCT-8 | ATCC | CCL-244 |
| **Experimental Models: Organisms/Strains** | | |
| Mouse: Ifngr1-/- (C57BL/6 background) | Jackson Laboratories | Cat# 003288 |
| Mouse: Nod scid gamma (NSG) | Jackson Laboratories | Cat# 005557 |
| **Oligonucleotides** | | |
| Primer: *C. parvum* GAPDH forward: CGGATGGCCATACCTGTGAG | [2] | N/A |
| Primer: *C. parvum* GAPDH reverse: GAAGATGCGCTGGGAACAAC | [2] | N/A |
| **Recombinant DNA** | | |
| Plasmid: TK-H3.1-3HA -Nluc-P2A-neo-TK | This paper | N/A |
| Plasmid: TK-H3.2-3HA -Nluc-P2A-neo-TK | This paper | N/A |
| Plasmid: CENH3-3HA-Nluc-P2A-neo | This paper | N/A |
| Plasmid: GBP-3HA-Nluc-P2A-neo | This paper | N/A |
| Plasmid: TK-3HA-CENH3-P2A-GBP-3Ty-Nluc-P2A-neo-TK | This paper | N/A |
| Plasmid: CENH3-smHA-Nluc-P2A-neo | This paper | N/A |
| Plasmid: pACT1:Cas9, U6:sgTK | [2] | Cat# 122852 |
| Plasmid: pACT1:Cas9, U6:sgCENH3 | This paper | N/A |
| Plasmid: pACT1:Cas9, U6:sgGBP | This paper | N/A |
| **Instruments, Software, and Algorithms** | | |
| GraphPad Prism 10 | GraphPad Software | <https://www.graphpad.com/> |
| QuantStudio Design & Analysis Software | Thermo Fisher Scientific | <https://www.thermofisher.com/us/en/home/global/forms/life-science/quantstudio-3-5-software.html> |
| FIJI (ImageJ) |  | <https://fiji.sc/> |
| Zeiss Observer Z1 inverted microscope with a Colibri LED illumination for multi-color epifluorescence | Zeiss | <https://www.zeiss.com/microscopy> |
| Hamamatsu ORCA-ER CCD Camera | Hamamatsu | <https://www.hamamatsu.com/us/en/product/cameras> |
| ZEN 2.5 software | Zeis | <https://www.zeiss.com/microscopy/en/products/software/zeiss-zen> |
| Zeiss Axioskop Mot Plus fluorescence microscope | Zeiss | <https://www.zeiss.com/microscopy> |
| AxioCam MRm monochrome digital camera and Axiovision software | Zeiss | <https://www.zeiss.com/microscopy/en/products/cameras> |
| Cytation 3 cell imaging multi-mode reader | Biotek | <https://www.agilent.com/en/product/microplate-instrumentation/microplate-readers/multimode-microplate-readers/biotek-cytation-hybrid-multimode-reader-1623197> |
| Veriti 96-well thermal cycler | Applied Biosystems | <https://www.thermofisher.com/order/catalog/product/4375305> |
| Qubit 4 Fluorometer | Invitrogen | <https://www.thermofisher.com/order/catalog/product/Q33226> |
| Alphafold 3 | Alphafold server | https://alphafoldserver.com/ |
| ChimeraX | UCSF ChimeraX | <https://www.cgl.ucsf.edu/chimerax/> |
| fastp v0.23.4 | Github | <https://github.com/OpenGene/fastp> |
| BWA-MEM v0.7.17 | BWA | <https://github.com/lh3/bwa> |
| picard v3.1.1 | Github | <https://broadinstitute.github.io/picard/> |
| SAMtools v1.7 | SAMtools | <http://samtools.sourceforge.net/> |
| ggplot2 | R | <https://www.rdocumentation.org/packages/ggplot2> |
| MEME v5.5.8 | MEME suite | <https://meme-suite.org/meme/tools/meme> |
| TBtools-II v2.311 | Github | <https://github.com/CJ-Chen/TBtools-II> |
