# Supplementary material for: Evidence for holocentric centromeres in the early branching apicomplexan parasite Cryptosporidium parvum
Source: PLoS Pathog. 2026 Oct 26;22(10):e1014708. doi: 10.1371/journal.ppat.1014708 (PMC13739221; doi:10.1371/journal.ppat.1014708)
Supplement: Supplementary file 10 [file ppat.1014708.s010.docx]

**S3 Table.** Oligonucleotides and plasmids used in this study.

| **Usage** | **Full Plasmid name** | **Oligo name** | **Sequence (5’ – 3’)** | **Purpose** | **Source** |
| --- | --- | --- | --- | --- | --- |
| Inserting an epitope tagged cgd3_2540 (H3.1) & cgd4_3220 (H3.2) into the TK locus | TK- cgd3_2540 (H3.1) -3HA-Nluc-P2A-neo-TK & TK- cgd4_3220 (H3.2)-3HA-Nluc-P2A-neo-TK | TK-cgd3_2540 BB Gib F | agattatgcctaatgttttttttaccaaataaatagctc | Amplify backbone from TK-GFP- Nluc-P2A-neo-TK plasmid for building TK- cgd3_2540 (H3.1) -3HA-Nluc-P2A-neo-TK by Gibson assembly | IDT |
|  |  | TK-cgd3_2540 BB Gib R | ttagaaacaaggtatgagtgatattattgctttaaag |  |  |
|  |  | TK-cgd4_3220 BB Gib F | agattatgccTAATGTTTTTTTTACCAAATAAATAGCTC | Amplify backbone from TK-GFP- Nluc-P2A-neo-TK plasmid for building TK- *cgd4_3220 (H3.2)-*3HA-Nluc-P2A-neo-TK by Gibson assembly |  |
|  |  | TK-cgd4_3220 BB Gib R | ttcaagcattggtatgagtgatattattgctttaaag |  |  |
|  |  | cgd3_2540-3xHA Gib F | gggtgaaagggctagcaagggctcgggc | Amplify pLinker-3xHA region from the CENH3-3HA-Nluc-P2A-neo plasmid for building TK- *cgd4_3220 (H3.2)-*3HA-Nluc-P2A-neo-TK by Gibson assembly |  |
|  |  | cgd3_2540-3xHA Gib R | AAAAAAAACATTAggcataatctggaacatcgtaaggatacg |  |  |
|  |  | cgd4_3220-3xHA Gib F | tgagaggtgagctagcaagggctcgggc | Amplify pLinker-3xHA region from the CENH3-3HA-Nluc-P2A-neo plasmid for building TK- *cgd4_3220 (H3.2)-*3HA-Nluc-P2A-neo-TK by Gibson assembly |  |
|  |  | cgd4_3220-3xHA Gib R | AAAAACATTAggcataatctggaacatcgtaaggatacg |  |  |
|  |  | TK-cgd3_2540 Gib F | cactcataccttgtttctaattagttcttcagatc | Amplify cgd3_2540 (H3.1) gene from *Cryptosporidium* DNA for building TK- *cgd3_2540 (H3.1)-*3HA-Nluc-P2A-neo-TK by Gibson assembly |  |
|  |  | TK-cgd3_2540 Gib R | ccttgctagccctttcacccctaattcttc |  |  |
|  |  | TK-cgd4_3220 Gib F | cactcataccaatgcttgaaagtacaagtgtgg | Amplify cgd4_3220 (H3.2) gene from *Cryptosporidium* DNA for building TK- *cgd4_3220 (H3.2)-*3HA-Nluc-P2A-neo-TK by Gibson assembly |  |
|  |  | TK-cgd4_3220 Gib R | ccttgctagctcacctctcaccacggatac |  |  |
|  |  | Cp-TK F2 | ACCATCTAACACAAAGAATTCAACA | To check correct gene insertion from DNA purified from mouse feces. | [1]; IDT |
|  |  | 3HA R | ggcataatctggaacatcgtaagg |  |  |
|  |  | Neo F1 | GCTGAAGAACTTGGTGGTGA |  |  |
|  |  | Cp TK-R | CCTACCATAAGCATCACCAGT |  |  |
| Epitope tagging of cgd4_2030 (CENH3) | CENH3 (cgd4_2030)-3HA-Nluc-P2A-neo | cgd4_2030 CENH3 BB Gib F | ctcggtacccGCTAGCAAGGGCTCGGGC | Amplify backbone from ABC-3HA-Nluc-P2A-neo plasmid for building CENH3 (cgd4_2030)-3HA-Nluc-P2A-neo by Gibson assembly | IDT |
|  |  | cgd4_2030 CENH3 BB Gib R | ccttgctagcGGGTACCGAGCTCGAATTCAC |  |  |
|  |  | cgd4_2030 CENH3 5' homology insert Gib F | ctcggtacccAACGAATAATAGAGCAATTAGTAG | Amplify cgd4_2030 (CENH3) 5' homology and 3'UTR from *Cryptosporidium c*DNA for building CENH3 (cgd4_2030)-3HA-Nluc-P2A-neo by Gibson assembly |  |
|  |  | cgd4_2030 CENH3 5' homology insert Gib R | ccttgctagcAATAAAGTCTCCATACCTGC |  |  |
|  |  | cgd4_2030 CENH3 3'UTR insert Gib F | atctgaacaaacggaggagtcttgttttttttaccaaataaatagctcc |  |  |
|  |  | cgd4_2030 CENH3 3'UTR insert Gib R | CCAACTCGGCCATCCACGCGccgctactaccgaccgttta |  |  |
|  | CENH3 Cas9 generation and PAM mutation | CENH3 gRNA2 | gTACAGGTATATAGTACTCCTgttttagagctagaaatagcaagt | Generate Cas9 backbone using DNA from the pCRISPR-Enolase-gRNA plasmid | IDT |
|  |  | U6 F | CCCAACACTTAACCTTTCAGT |  |  |
|  |  | CENH3 PAM MUT gRNA2 F1 | agtactcctagttcaatttaattca | Mutate the PAM sequence in the CENH3 tagging plasmid so that it is not recognized by the Cas9 |  |
|  |  | CENH3 PAM MUT gRNA2 R1 | atatacctgtaatatatgttaattttagtt |  |  |
|  | Gene insertion confirmation | CenH3 5'UTR check F2 | AGTAGCCCCATTAGCCCAAG | To check correct gene insertion from DNA purified from mouse feces. | [1]; IDT |
|  |  | 3HA-R | ggcataatctggaacatcgtaagg |  |  |
|  |  | CenH3 3'UTR check R | GGCGGGGATAATTCATGTTT |  |  |
|  |  | NeoF1 | GCTGAAGAACTTGGTGGTGA |  |  |
| Epitope tagging of cgd1_3530 (GBP1) | GBP (cgd1_3530)-3HA-Nluc-P2A-neo | LacZ F2 | GGGGATCCTCTAGAGTCGAC | Generate AmpR region of the plasmid backbone from the Enolase-3HA-Nluc-neo-gRNA2 plasmid | [2]; IDT |
|  |  | LacZ R1 | GGGTACCGAGCTCGAATT |  |  |
|  |  | Linker F1 | gctagcaagggctcgggc | Generate the selection casette region of the plasmid backbone from the Enolase-3HA-Nluc-neo-gRNA2 plasmid |  |
|  |  | Neo R2 | caattgtcagaagaattcgt |  |  |
|  |  | Gbp 5' homo Gib F1 | ctcggtacccTGAATTAAATAATACTACATTACTTGATC | Amplify cgd1_3530 (GBP1) 5' homology and 3'UTR from *Cryptosporidium* gDNA for building GBP1 (cgd1_3530)-3HA-Nluc-P2A-neo by Gibson assembly | IDT |
|  |  | Gbp 5' homo Gib R1 | gagcccgagcccttgctagcCTGGTCGATTCTAACCAATA |  |  |
|  |  | Gbp 3' UTR Gib F1 | ctgacaattgATTAATTCAAAATTACTTTAAAGTCAATTTTTTG |  |  |
|  |  | Gbp 3' UTR Gib R1 | cttgctagcAATTGAACTTTCTATTTTTTTTCATTG |  |  |
|  | GBP Cas9 generation and PAM mutation | Tracer RNA F1 | gttttagagctagaaatagcaag | Generate Cas9 backbone using DNA from the pCRISPR-Enolase-gRNA plasmid | [2]; IDT |
|  |  | U6 F | CCCAACACTTAACCTTTCAGT |  |  |
|  |  | cgd1_3530_gRNA1_105 Linker | ctgaaaggttaagtgttgggGAGGCTATGGAATGAGAACAgttttagagctagaaatagc | gRNA linker sequence for Gibson assembly with Cas9 backbone | IDT |
|  |  | GBP PAM MUT gRNA1 F1 | ATGAGAACAAAGACTCATGCT | Mutate the PAM sequence in the GBP1 tagging plasmid so that it is not recognized by the Cas9 |  |
|  |  | GBP PAM MUT gRNA1 R1 | TCCATAGCCTCTGTTATTCCA |  |  |
|  | Gene insertion confirmation | Gbp Insert F1 | GAGCTATCAATGAATTAAATAATAC | To check correct gene insertion from DNA purified from mouse feces. | [1]; IDT |
|  |  | pEno R | tagagtttggtgtgcaggcg |  |  |
|  |  | NeoF1 | GCTGAAGAACTTGGTGGTGA |  |  |
|  |  | Gbp Insert R2 | GAGTGGTGTTTTACCATGGAATTGATATCATTATC |  |  |
| Inserting an epitope tagged cgd4_2030 (CENH3) & cgd1_3530 (GBP) into the TK locus | Generating TK-3HA-CENH3-P2A-GBP-3TY-TK tagging plasmmid | CenH3-Gbp Backbone F | GGTCGGTAGTAGCGGaggtggggaaactaaatata | Amplify backbone from TK-3HA-CENH3-Nluc-P2A-neo-TK plasmid (not featured in MS) for building TK-3HA-CENH3-P2A-GBP-3TY-TK by Gibson assembly | IDT |
|  |  | CenH3-Gbp Backbone R | ACAACCAAAATATTCggtatgagtgatattattgc |  |  |
|  |  | CenH3 5'UTR-3xHA-pLinker F | aatatcactcataccGAATATTTTGGTTGTTTAATTAAGATATTATTCATGCATGAAACG | Amplify 3HA-pLinker region from TK-3HA-CENH3-Nluc-P2A-neo-TK plasmid (not featured in MS) for gibson assembly |  |
|  |  | CenH3 5'UTR-3xHA-pLinker R | TTTAGAACGAGCCATcagctgggtcgagcccga |  |  |
|  |  | CenH3 CDS F | ggctcgacccagctgATGGCTCGTTCTAAAACTTC | Amplify CENH3 gene from *Cryptosporidium* cDNA for gibson assembly |  |
|  |  | CenH3 CDS R | agtagctccgcttccAATAAAGTCTCCATACCTGC |  |  |
|  |  | Gbp CDS F | gagaaccctggacctATGGTATCTGATAAAAACTGC | Amplify GBP gene from *Cryptosporidium* gDNA for gibson assembly |  |
|  |  | Gbp CDS R | cgagcccttgctagcCTGGTCGATTCTAACCAATATC |  |  |
|  |  | pLinker-Tyx3 F | GTTAGAATCGACCAGgctagcaagggctcgggc | Amplify pLinker-Tyx3 region from GP-mAID-3HA-TIR1-Nluc-P2A-neo plasmid for gibson assembly | [3]; IDT |
|  |  | pLinker-Tyx3 R | TTGGTAAAAAAAACATTAGTCGAGCGGGTCCTGG |  |  |
|  |  | CenH3 3'UTR F | GGACCCGCTCGACTAATGTTTTTTTTACCAAATAAATAGCTCCTTATTTGC | Amplify CENH3 3'UTR region from TK-3HA-CENH3-Nluc-P2A-neo-TK plasmid (not featured in MS) for gibson assembly |  |
|  |  | CenH3 3'UTR R | ttagtttccccacctCCGCTACTACCGACCGTTTATTG |  |  |
|  |  | P2A Linker | AGACTTTATTggaagcggagctactaacttcagcctgctgaagcaggctggagacgtggaggagaaccctggacctATGGTATCTG | P2A Linker gBlock for for gibson assembly |  |
|  | Gene insertion confirmation | Cp-TK F2 | ACCATCTAACACAAAGAATTCAACA | To check correct gene insertion from DNA purified from mouse feces. | [1]; IDT |
|  |  | 3HA R | ggcataatctggaacatcgtaagg |  |  |
|  |  | Neo F1 | GCTGAAGAACTTGGTGGTGA |  |  |
|  |  | Cp TK-R | CCTACCATAAGCATCACCAGT |  |  |
| Epitope tagging of cgd4_2030 (CENH3) (spaghetti monster HA) | CENH3 (cgd4_2030)-smHA-Nluc-P2A-neo | Linker-F1 | gctagcaagggctcgggc | Amplify spaghetti monster tag region from p2870-smHA-Nluc-P2A-neo | [1]; [3]; IDT |
|  |  | AldoUTR_R | ggcgcgccaaataaagtaaagtttatcg |  |  |
|  |  | Aldo 3UTR-F | atgcatcttcatttagtatcttaggt | Amplify CENH3 (cgd4_2030)-3HA-Nluc-P2A-neo without the 3HA tag |  |
|  |  | pLinker-R2 | cagctgggtcgagcccga |  |  |
|  | Gene insertion confirmation | CenH3 5'UTR check F2 | AGTAGCCCCATTAGCCCAAG | To check correct gene insertion from DNA purified from mouse feces. | [1]; IDT |
|  |  | pLinker-R2 | cagctgggtcgagcccga |  |  |
|  |  | CenH3 3'UTR check R | GGCGGGGATAATTCATGTTT |  |  |
|  |  | NeoF1 | GCTGAAGAACTTGGTGGTGA |  |  |

**References:**

1.	Wilke G, Ravindran S, Funkhouser-Jones L, Barks J, Wang Q, VanDussen KL, et al. Monoclonal Antibodies to Intracellular Stages of Cryptosporidium parvum Define Life Cycle Progression In Vitro. mSphere. 2018;3(3). Epub 20180530. doi: 10.1128/mSphere.00124-18. PubMed PMID: 29848759; PubMed Central PMCID: PMCPMC5976880.

2.	Kimball A, Funkhouser-Jones L, Huang W, Xu R, Witola WH, Sibley LD. Mendelian segregation and high recombination rates facilitate genetic analyses in Cryptosporidium parvum. PLoS Genet. 2024;20(6):e1011162. Epub 20240617. doi: 10.1371/journal.pgen.1011162. PubMed PMID: 38885280; PubMed Central PMCID: PMCPMC11213348.

3.	Xu R, Beatty WL, Greigert V, Witola WH, Sibley LD. Multiple pathways for glucose phosphate transport and utilization support growth of Cryptosporidium parvum. Nat Commun. 2024;15(1):380. Epub 20240109. doi: 10.1038/s41467-024-44696-3. PubMed PMID: 38191884; PubMed Central PMCID: PMCPMC10774378.
